# Constraint and Allometric Diversification in a Simplified Neck: Shape Evolution of the Atlas in Hyloidea (Anura)

**DOI:** 10.3390/biology15141200

**Published:** 2026-07-20

**Authors:** Henrique Folly, Jéssica Fratani, Virginia Abdala, María Laura Ponssa

**Affiliations:** 1 Área Herpetología, Unidad Ejecutora Lillo (UEL), FML-CONICET, San Miguel de Tucumán 4000, Tucumán, Argentina; follyhenrique@gmail.com (H.F.); jessicafratani@gmail.com (J.F.); 2 Cátedra de Biología General, Facultad de Ciencias Naturales e IML, UNT, San Miguel de Tucumán 4000, Tucumán, Argentina; 3 Instituto de Biodiversidad Neotropical (IBN), UNT-CONICET, Yerba Buena 4107, Tucumán, Argentina

**Keywords:** anura, ecomorphology, geometric morphometrics, vertebral column

## Abstract

The anuran atlas represents one of the most structurally simplified cervical systems among tetrapods, consisting of a single vertebra that must simultaneously support the head, permit mobility, and withstand mechanical loads generated during locomotion and feeding. This unique configuration provides a compelling framework for investigating how structural constraint, allometry, and ecology interact to shape morphological evolution. Here, we examine atlas shape diversification across Hyloidea using geometric morphometrics and phylogenetic comparative methods on 421 specimens representing 133 species. We quantified shape variations in dorsal and ventral views and tested the relative contributions of body size, phylogenetic history, microhabitat, and locomotor mode to atlas morphology. Atlas shape exhibited significant phylogenetic signals and was predominantly structured by allometric scaling, with the atlas size (centroid size) explaining a significant proportion of variation in both anatomical views, particularly ventrally. Morphological differences were concentrated in atlas width and relative proportions, indicating that diversification occurs primarily through size-dependent remodeling rather than through major structural reorganization. In contrast, ecological variables explained little independent variation in atlas shape, although microhabitat modulated allometric trajectories in dorsal and ventral views. Together, these results indicate that atlas evolution in hyloid frogs is governed chiefly by size-related biomechanical demands operating within a phylogenetically structured and developmentally constrained system. More broadly, our findings suggest that extreme cervical simplification channels morphological diversification toward allometric modification, limiting the extent to which ecological specialization can drive anatomical divergence.

## Simple Summary

Frogs have one of the simplest necks among terrestrial vertebrates, made up of a single bone, called the atlas, that supports the head, allows it to move, and withstands the forces produced during jumping and feeding. This simple structure makes frogs a useful example for understanding how physical limits, body size, and lifestyle together shape the way animal bodies evolve. In this study, we examined the atlas shape using digital images from 133 frog species and statistical methods that also take into account how closely related the species are. We found that larger frogs have proportionally different bones, especially in width, and this was the main factor explaining shape differences, more so than the environment where frogs live or how they move. This means that, rather than undergoing major structural changes, the bone evolves mainly through adjustments tied to body size. These findings suggest that when a body structure is already very simple, its evolution becomes limited mostly to this kind of size-related change, restricting how much the environment and lifestyle can shape its form.

## 1. Introduction

The neck plays a central role in the functional integration of the vertebrate body, mediating the mechanical and structural connection between the head and the trunk [1,2]. From an evolutionary perspective, the origin of a functional neck represents one of the major anatomical transformations associated with the transition from fishes to tetrapods. This shift involved extensive musculoskeletal reorganization at the head–trunk interface, replacing the rigid connection between the head and the pectoral girdle present in fishes with a mobile articulation that allowed the head to move independently from the trunk [3]. In most tetrapods, this region is composed of multiple cervical vertebrae that collectively support the head, allow controlled rotation, and contribute to a wide range of behaviors, including feeding, stabilization during locomotion, and other ecologically relevant actions, as it enables precise movements during these activities (e.g., [4,5,6,7,8,9,10,11,12]). By enabling independent head movements, the neck also facilitates orientation toward sensory stimuli and contributes to key biological functions such as feeding, respiration, and vocalization, given its close association with structures such as the hyoid apparatus, pharynx, and larynx [2].

Variations in neck morphology across vertebrates may arise through changes in cervical vertebral number or through modifications in the size and proportions of individual vertebrae [13]. In many lineages, neck elongation reflects coordinated changes among serially repeated cervical elements in response to functional demands [14,15,16]. Amphibians, however, exhibit a markedly simplified condition in which the cervical region is reduced to a single vertebra [15]. This condition is not merely a reduction in the ancestral tetrapod neck but likely reflects a secondary evolutionary reorganization of the craniovertebral boundary associated with shifts in axial patterning during lissamphibian evolution [17]. Consequently, the anuran atlas represents the outcome of a profound reconfiguration of the head–trunk interface rather than simply a reduced cervical element.

In anurans, the neck is represented solely by the atlas, a specialized, ring-shaped vertebra lacking ribs and an axis, and articulating directly with the occipital condyles of the skull [18]. This unique configuration establishes a highly constrained craniovertebral articulation, in which a single element must simultaneously provide support, mobility, and mechanical stability. Despite its functional centrality, the atlas has received comparatively little attention in macroevolutionary and allometric studies, particularly in amphibians [13]. While patterns of skull or column allometry have been explored in frogs [19,20,21,22,23], formal assessments of cervical allometric trajectories and their ecological correlates remain scarce in this group.

Despite extensive advances in anuran ecomorphology, research has largely focused on cranial diversity [20,24], appendicular morphology (e.g., [25,26,27,28,29,30,31,32,33,34,35,36,37]), and the vertebral column [21], particularly its posterior region, with special emphasis on its functional integration with the pelvic girdle and its importance for saltatory locomotion. This body of work has substantially advanced our understanding of the anatomical correlates of jumping performance and ecological diversification. However, the potential role of atlas morphology in ecological diversification remains poorly understood. This omission is striking given that the atlas constitutes the sole vertebral element connecting the axial skeleton to the skull in anurans. As such, it is strategically positioned to mediate force transmission between the head and trunk, stabilize the skull during landing and locomotion, and regulate head mobility during feeding and other ecologically relevant behaviors [6,38,39].

Anurans of the clade Hyloidea constitute a particularly powerful system for testing ecomorphological hypotheses, given their remarkable morphological diversity and extensive ecological radiation. Species occupy a wide range of microhabitats and exhibit distinct locomotor modes, from arboreal climbing to terrestrial jumping and aquatic propulsion [33], offering a robust framework to assess whether variations in atlas morphology reflect ecological specialization, biomechanical demands, or size-related scaling patterns [36]. This framework is further strengthened by the substantial body-size diversity represented in our dataset, spanning more than an order of magnitude in vertebral column length, from *Allophryne ruthveni* (6.5 mm) to *Leptodactylus laticeps* (70.7 mm), providing an ideal context to investigate how atlas morphology scales with size.

In this study, we investigate the factors shaping atlas morphological variations across hyloid anurans by testing the relative contributions of ecology, body size, and phylogenetic history to atlas shape diversification. Specifically, we assess whether atlas morphology varies among ecological categories, including microhabitat and locomotor mode, while accounting for allometric scaling and shared ancestry. We predict that if ecological specialization influences atlas evolution, species occupying contrasting ecological niches should differ in atlas shape and/or allometric trajectories. Conversely, if structural and developmental constraints or phylogenetic conservatism predominate, atlas morphology should be explained primarily by body size and shared ancestry.

## 2. Material and Methods

### 2.1. Material and Data Collection

We analyzed 421 adult specimens representing 133 species from 19 anuran families of the clade Hyloidea [40]. The examined specimens are housed in the herpetological collections of the Fundación Miguel Lillo, San Miguel de Tucumán, Argentina (FML, including material cataloged as CU and MCV); Museo Argentino de Ciencias Naturales “Bernardino Rivadavia”, Buenos Aires, Argentina (MACN, including material cataloged as BB, CENAI, CFBH, DB, JF and LGE); Instituto de Investigación Biológica del Paraguay, Asunción, Paraguay (IIBP); Coleção Zoológica de Referência da Universidade Federal de Mato Grosso do Sul, Campo Grande, Brazil (ZUFMS); and Museu de Zoologia João Moojen, Universidade Federal de Viçosa, Viçosa, Brazil (MZUFV). Additional specimens were obtained as micro-computed tomography (µCT) scans through the MorphoSource platform (www.morphosource.org, accessed on 16 March 2026) (acronyms: CAS = California Academy of Sciences; CM = Carnegie Museum of Natural History; KUH or KU = University of Kansas; MVZ = Museum of Vertebrate Zoology, University of California; UCM = University of Colorado Museum of Natural History; UF = University of Florida). Detailed information for all specimens examined is provided in Supplementary Material S1.

Some specimens were already available as skeletal preparations in the collections, whereas others were cleared and stained following a protocol adapted from Taylor & Van Dike [41]. In these preparations, cartilaginous structures were stained with Alcian blue, while mineralized and ossified tissues were stained with Alizarin Red S. Sex information was unavailable for specimens deposited as skeletal preparations. Taxonomic nomenclature follows Frost [42]. The general terminology for the atlas follows Lynch [43].

**Figure 1:**
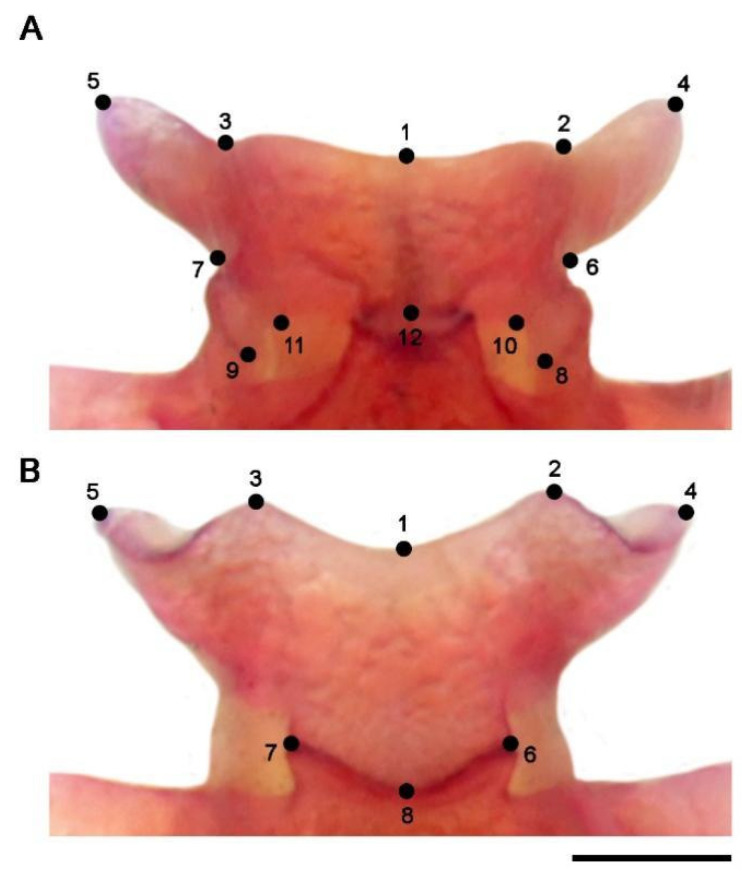
Landmark configuration in dorsal (**A**) and ventral (**B**) views of the atlas used for the geometric morphometric analyses. For a detailed description of the landmarks, see Table 1. Specimen shown in the photograph: *Scinax fuscovarius* (FML 29825). Scale bar = 1 mm.

Photographs of the atlas were taken with a Canon 80D camera equipped with a 100 mm macro lens, ensuring standardized capture conditions across all specimens. Each atlas was positioned perpendicularly to the optical axis of the lens and photographed in dorsal and ventral views, with a scale bar included in all images (Figure 1A,B). For specimens obtained via computed tomography, a 2D image was generated to ensure consistency with the remaining samples. Because the objectives of this study focused on variations in the dorsal and ventral shape of the atlas, and because most specimens consisted of cleared-and-stained museum material, we adopted a standardized two-dimensional geometric morphometric approach. This strategy enabled the inclusion of a much broader taxonomic sample while maintaining methodological consistency across all specimens, including those available as µCT scans, which were projected into comparable 2D views. A total of 133 species were included in the analyses; of these, 132 were examined in dorsal view and 118 in ventral view. The reduced ventral sample reflects the fact that some specimens were not disarticulated, precluding visualization of the ventral surface of the atlas. Specimens with damaged bones or evident morphological anomalies were excluded from the analyses. Landmark digitization was performed by a single operator (HF) using the StereoMorph R package [44]. Landmarks were selected based on their anatomical homology and their ability to accurately describe the outer borders of the atlas, ensuring repeatability across all analyzed specimens. Landmark selection followed Adler et al. [21], with additional reference points incorporated to capture structures of particular relevance to this study (Figure 1A,B). For the dorsal view, 12 anatomically homologous landmarks were placed, including five bilaterally symmetric pairs (landmarks 2–11) and two midline landmarks (1, 12). For the ventral view, eight landmarks were positioned, including three bilaterally symmetric pairs (landmarks 2–7) and two midline landmarks (1, 8). A detailed description of the landmarks is provided in Figure 1 and Table 1.

### 2.2. Statistical Analyses

Atlas shape variation was analyzed using the *geomorph* and ‘RRPP’ packages [45,46,47,48] in the R programming language (v.4.1.2). Landmark coordinates were subjected to Generalized Procrustes Analysis (GPA) using *gpagen* function to remove non-shape variations from the landmark data by translating, scaling, and rotating configurations to a common coordinate system [48,49]. Atlas size was quantified by the centroid size (CS). Because multiple specimens were digitized per species, species mean shapes were computed using *coords.subset* to separate configurations by taxon and *mshape* to calculate the mean shape of each species [48]. Mean centroid size per species was calculated as the arithmetic mean of CS values across all specimens of that species. Then, bilateral symmetry was assessed using the *bilat.symmetry* function, with 1000 permutations using the residual randomization procedure [48]. All subsequent analyses were conducted using the symmetric shape component.

The relationship between shape and atlas centroid size (allometry) was assessed by multivariate regression of shape on the natural logarithm of centroid size (log CS) using two approaches: (i) static allometry by Ordinary Least Squares (OLS) with the *procD.lm* function, without phylogenetic correction; and (ii) evolutionary allometry by Phylogenetic Generalized Least Squares (PGLS) with the *procD.pgls* function, incorporating a phylogenetic covariance matrix [50,51]. Statistical significance was evaluated by residual randomization with 1000 iterations. Allometric trajectories were visualized using the *plotAllometry* function (method RegScore), which projects each species’ shape onto the allometric regression vector, generating a univariate score (Regression Score) summarizing size-associated shape variations [48]. Points were colored by microhabitat categories and symbols representing locomotor mode categories (detailed below). Regression lines per group were added manually to visualize the allometric trajectory of each category.

Phylogenetic signals were assessed separately for shape and size. For Procrustes coordinates (multivariate data), the *physignal.z* function was used with the parameter lambda = ‘burn’, which estimates the effect size (Z-score) and optimizes Pagel’s lambda via 1000 permutations [52,53]. For centroid size (univariate variable), the *physignal* function was used to estimate Blomberg’s K statistic [54].

To visualize the distribution of shape variation in a phylogenetic context, a Principal Component Analysis with phylogenetic branches projected into ordination plots (phylomorphospace) was performed using the *gm.prcomp* function [55]. This approach projects Procrustes shape variables for species into a multivariate shape space and overlays phylogenetic connections, enabling visualization of shape evolution along lineages and identification of potential morphological convergences or divergences [56,57]. Plots were produced with species colored according to microhabitat categories and symbols representing locomotor mode categories. The extreme shapes at the minimum and maximum of PC1 and PC2 were visualized as thin-plate spline deformation grids using *plotRefToTarget* function, comparing each extreme to the overall mean shape, to identify which anatomical regions of the atlas vary along each principal component [48].

The phylogeny was obtained from Portik et al. [40], currently one of the most comprehensive phylogenies available for amphibians. The complete tree was imported into R using the *read.tree* function of the ‘*ape*’ package [58] and pruned to include only the species present in the morphometric datasets using *keep.tip*. The pruned tree was edited in Mesquite [59] for species name standardization, correction and inclusion of additional taxa when necessary. The species not included in Portik et al. [40] were added based on the hypotheses of Guillory et al. [60] for *Hyloxalus*, de Sá et al. [61] for *Leptodactylus*, Lourenço et al. [62] for *Physalaemus*, Faivovich et al. [63] and Ferraro [64] for *Pleurodema*, Pereyra et al. [65] for *Rhinella*, Barrionuevo [66] and Sáez et al. [67] for *Telmatobius*, and Guayasamin et al. [68] for *Vitreorana*. Branch lengths were estimated using the *compute.brlen* function of the *‘ape’* package [58]. Remaining polytomies were randomly resolved using the *multi2di* function available in the *‘ape’* R library [58]. The correspondence between morphometric data and phylogeny was verified by ensuring the same species order in both objects prior to each analysis.

Microhabitat and locomotor mode for each species were compiled from the specialized literature (e.g., [32,35,69]) combined with field observations. Species were classified into four microhabitat categories: aquatic, arboreal, semi-aquatic, and terrestrial; and into four locomotor mode categories: hopper, jumper, swimmer, and walker. Categories were assigned following the criteria proposed by Ponssa et al. [70]. Data were integrated into the morphometric dataset through a metadata table, retaining only species present in both the phylogeny and the shape data. To assess the influence of ecological variables on atlas shape, PGLS models were fitted using the *procD.pgls* function, with log CS as a covariate and microhabitat or locomotor mode as categorical factors. For each ecological variable, an additive model (shape ~ log(CS) + factor) and an interaction model (shape ~ log(CS) × factor) were compared using nested model ANOVA with RRPP (1000 iterations) to evaluate whether allometric trajectories differ among groups (heterogeneous allometry). Pairwise comparisons among groups were performed using the *pairwise* function of the ‘RRPP’ package [45].

## 3. Results

### 3.1. Allometry

**Figure 2:**
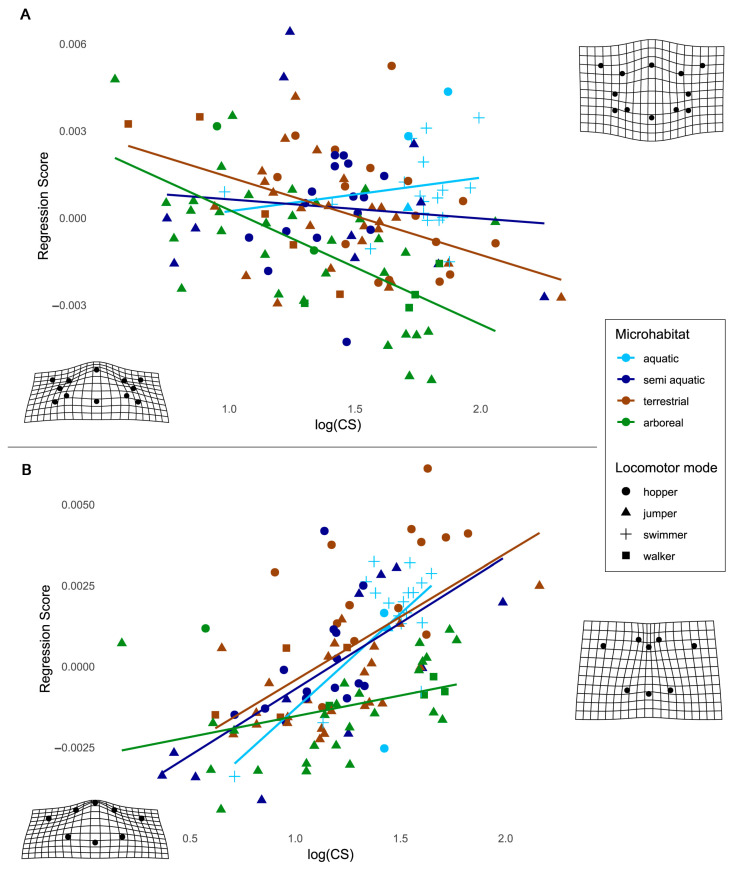
Allometric trajectories of the atlas in dorsal (**A**) and ventral (**B**) views. The *X*-axis represents the natural logarithm of centroid size (log CS) as a proxy for body size, and the *Y*-axis represents the Regression Score (RegScore method), a univariate score summarizing size-associated shape variation. Each point corresponds to a species. Colors represent habitat categories: aquatic (blue), semi-aquatic (dark blue), terrestrial (brown), and arboreal (green). Symbols represent locomotor mode categories: circle (hopper), triangle (jumper), plus sign (swimmer), and square (walker). Regression lines represent the allometric trajectory of each microhabitat category. Deformation grids represent the mean shape of the minimum (left) and maximum (right) predicted shapes for each atlas view.

For the dorsal atlas surface, static allometry estimated by OLS indicated that centroid size (log CS) explained 3.3% of shape variations (R^2^ = 0.033; F = 4.473; *p* = 0.006), reflecting a statistically significant but low-magnitude allometric relationship. After incorporating phylogenetic structure through PGLS, the relationship remained significant and increased in magnitude (R^2^ = 0.060; F = 8.348; *p* = 0.001). Microhabitat groups showed a pattern of distinct regression slopes that converge at smaller atlas sizes and progressively diverge as body size increases (Figure 2A). At smaller atlas sizes, all groups occupy similar regions of dorsal atlas shape space, suggesting comparable shape configurations regardless of microhabitat. As atlas size increases, however, the trajectories diverge markedly. Arboreal species exhibit the steepest negative allometric trajectory, as atlas size increases, with dorsal atlas shape shifting toward increasingly negative regression scores, while aquatic species display a comparatively flat or slightly positive trend (Figure 2A and Figure S1). See Section 3.4 for statistical comparisons of allometric slopes.

For the ventral atlas surface, static allometry was substantially stronger than that observed dorsally (R^2^ = 0.269; F = 42.869; *p* = 0.001), with atlas size accounting for over one quarter of ventral shape variations. While exploring evolutionary allometry via PGLS, the allometric relationship remained significant (R^2^ = 0.188; F = 27.014; *p* = 0.001). A consistent positive relationship between log CS and the Regression Score was clearly visible across all microhabitat groups; however, the slopes showed different patterns among groups (Figure 2B). Semi-aquatic and terrestrial species showed the steepest positive trajectories, with their regression lines running nearly parallel throughout the size range. Aquatic species followed a moderately positive slope, occupying an intermediate position. Arboreal species exhibited the shallowest trajectory among all groups, such that specimens with larger atlas size showed comparatively little change in ventral atlas shape relative to smaller ones. A pattern of size-amplified divergence was again observed: at smaller atlas sizes, all groups converged toward similar negative regression scores, while at larger atlas sizes the trajectories diverged substantially, most notably between aquatic and arboreal species (Figure 2B and Figure S2). See Section 3.4 for statistical comparisons of allometric slopes.

### 3.2. Phylogenetic Signal

The phylogenetic signal for dorsal atlas shape was highly significant (Z = 7.090; *p* = 0.001; λ = 1.0; K = 0.04). The λ value indicates that shape variation retains a phylogenetic covariance structure consistent with Brownian expectations, such that closely related species tend to resemble one another. However, the low K value (0.04) suggests that the magnitude of resemblance among relatives is weaker than expected under a pure Brownian motion model. For size, the phylogenetic signal was also significant (K = 0.058; *p* = 0.001), although of low magnitude, indicating relatively weak phylogenetic structuring of centroid size variation within Hyloidea. For the ventral atlas surface, the phylogenetic signal for shape was likewise highly significant (Z = 7.740; *p* = 0.001; λ = 1.0; K = 0.046), with a magnitude only slightly greater than that observed for the surface. Phylogenetic signal for size was also significant and comparable to dorsal values (K = 0.064; *p* = 0.001).

### 3.3. Phylomorphospace

**Figure 3:**
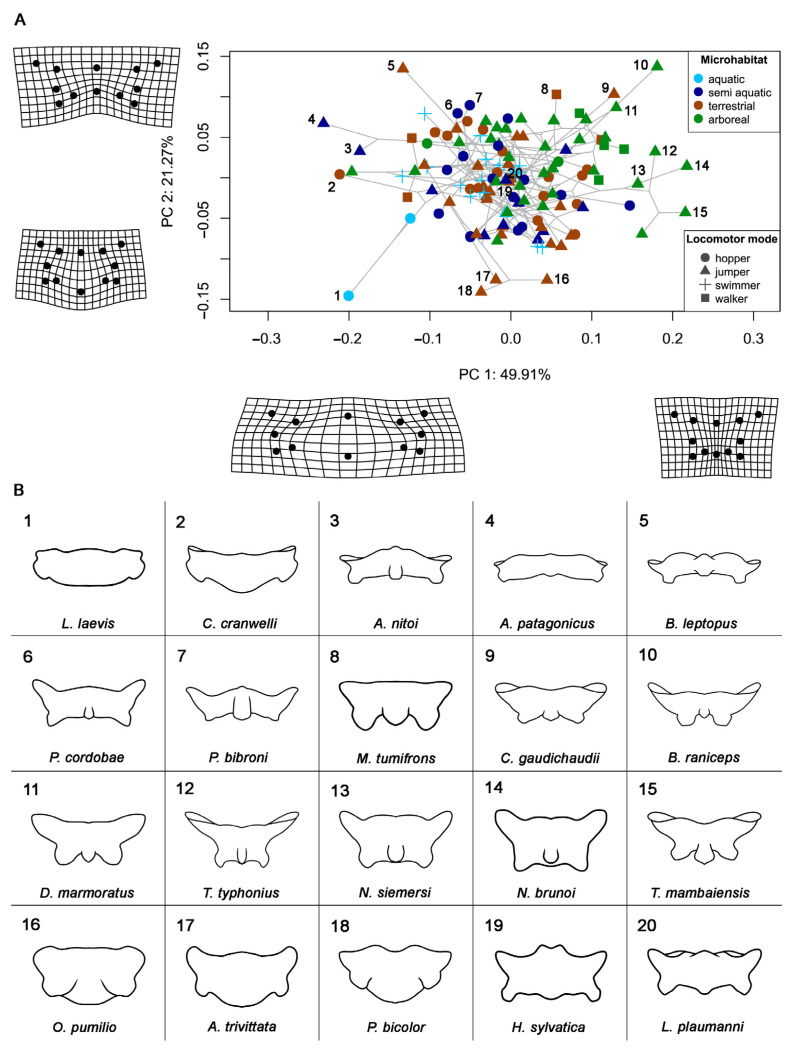
(**A**) Phylomorphospace of atlas shape in dorsal view based on geometric morphometric data. Deformation grids illustrate the shape changes associated with the minimum and maximum extremes of PC1 and PC2. Each point represents a species, connected by thin gray lines reflecting phylogenetic structure. Colors represent habitat categories: aquatic (blue), semi-aquatic (dark blue), terrestrial (brown), and arboreal (green). Symbols represent locomotor mode categories: circle (hopper), triangle (jumper), plus sign (swimmer), and square (walker). (**B**) Dorsal view illustrations of selected species representing the range of atlas shape variations across the morphospace, numbered as in: 1. *Lepidobatrachus laevis*, 2. *Ceratophrys cranwelli*, 3. *Atelognathus nitoi*, 4. *Atelognathus patagonicus*, 5. *Batrachyla leptopus*, 6. *Pleurodema cordobae*, 7. *Pleurodema bibroni*, 8. *Melanophryniscus tumifrons*, 9. *Crossodactylus gaudichaudii*, 10. *Boana raniceps*, 11. *Dendropsophus marmoratus*, 12. *Trachycephalus typhonius*, 13. *Nyctimantis siemersi*, 14. *Nyctimantis brunoi*, 15. *Trachycephalus mambaiensis*, 16. *Oophaga pumilio*, 17. *Ameerega trivittata*, 18. *Phyllobates bicolor*, 19. *Hylorina sylvatica*, 20. *Leptodactylus plaumanni*.

**Figure 4:**
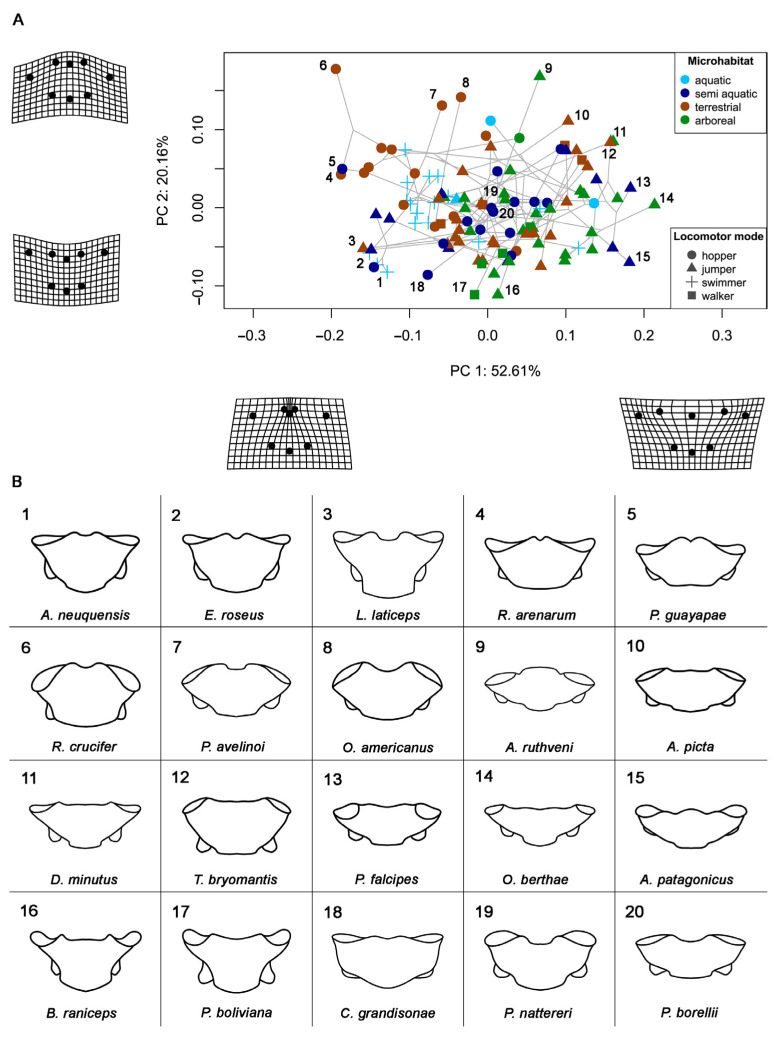
(**A**) Phylomorphospace of atlas shape in ventral view based on geometric morphometric data. Deformation grids illustrate the shape changes associated with the minimum and maximum extremes of PC1 and PC2. Each point represents a species, connected by thin gray lines reflecting phylogenetic structure. Colors represent habitat categories: aquatic (blue), semi-aquatic (dark blue), terrestrial (brown), and arboreal (green). Symbols represent locomotor mode categories: circle (hopper), triangle (jumper), plus sign (swimmer), and square (walker). (**B**) Ventral view illustrations of selected species representing the range of atlas shape variations across the morphospace, numbered as in: 1. *Alsodes neuquensis*, 2. *Eupsophus roseus*, 3. *Leptodactylus laticeps*, 4. *Rhinella arenarum*, 5. *Pleurodema guayapae*, 6. *Rhinella crucifer*, 7. *Proceratophrys avelinoi*, 8. *Odontophrynus americanus*, 9. *Allophryne ruthveni*, 10. *Ameerega picta*, 11. *Dendropsophus minutus*, 12. *Thoropa bryomantis*, 13. *Pseudopaludicola falcipes*, 14. *Ololygon berthae*, 15. *Atelognathus patagonicus*, 16. *Boana raniceps*, 17. *Phyllomedusa boliviana*, 18. *Chaltenobatrachus grandisonae*, 19. *Physalaemus nattereri*, 20. *Pleurodema borellii*.

**Figure 5:**
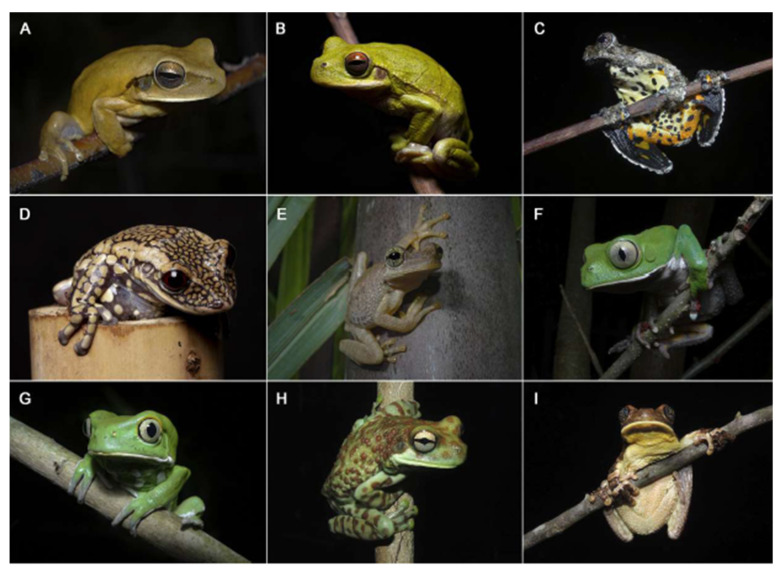
Examples of arboreal anuran species exhibiting dorsoventral and lateral head movements while navigating vegetation, highlighting the remarkable head mobility and repositioning associated with locomotion on complex three-dimensional substrates. (**A**) *Boana raniceps*, (**B**) *B*. *riojana*, (**C**) *Dendropsophus marmoratus*, (**D**) *Nyctimantis pomba*, (**E**) *Osteocephalus taurinus*, (**F**) *Phyllomedusa burmeisteri*, (**G**) *P*. *sauvagii*, (**H**) *Trachycephalus cunauaru*, and (**I**) *T*. *typlonius*. Photos: Folly, H.

In the principal component analysis (PCA) of the dorsal atlas surface, PC1 and PC2 explained over 70% of the observed interspecific variability (Figure 3A and Figure S3). PC1 reflects variations in the overall width of the atlas: species with negative scores exhibit a wider atlas with laterally expanded margins and a relatively flattened central region (e.g., first-row species, Figure 3B), whereas toward the positive extreme, the atlas becomes progressively narrower and more elongated along the anteroposterior axis, with contracted lateral margins and a more prominent central region (e.g., third-row species, Figure 3B). PC2 captures variations in the shape of the anterior and posterior margins: species with negative scores exhibit a convex posterior margin of the neural arch (e.g., Figure 3B-1, 16, 17, 18), whereas at the positive extreme, this region becomes concave (e.g., Figure 3B-5, 8, 9, 10). Visual inspection of the phylomorphospace revealed broad overlap among microhabitat groups along both axes, although a tendency toward separation between aquatic and arboreal species was perceptible. Aquatic species tended to concentrate in the central to slightly negative region of PC1 with more neutral PC2 scores, whereas arboreal species showed a broader distribution with a tendency toward central to positive PC1 values. This tendency, however, was not confirmed statistically by pairwise comparisons (see Microhabitat and Locomotor Mode results below). Terrestrial and semi-aquatic species occupied largely overlapping central positions in the morphospace, showing no clear tendency toward either extreme of PC1 or PC2.

For the ventral atlas surface, PC1 and PC2 explained over 71% of interspecific variability (Figure 4A and Figure S4). PC1 captures variations in cotyle projection and overall atlas width: at the negative extreme, the ventral atlas exhibits more developed occipital cotyles positioned closer to one another (e.g., first-row species, Figure 4B), whereas toward the positive extreme the atlas becomes wider with less pronounced cotyles that are more widely spaced (e.g., third-row species, Figure 4B). PC2 captures variations in margin shape: at the negative extreme the anterior region of the ventral atlas is concave (e.g., Figure 4B-15, 16, 17, 18), becoming convex at the positive extreme (e.g., Figure 4B-7, 8, 9, 10). Inspection of the ventral phylomorphospace also revealed a tendency toward separation between aquatic and arboreal species along PC1. Aquatic species tended to concentrate in the central to slightly negative region of PC1, whereas arboreal species showed a tendency toward central to positive PC1 values. This distributional tendency, however, was not confirmed statistically by pairwise comparisons (see Microhabitat and Locomotor Mode results below). Terrestrial and semi-aquatic species occupied largely overlapping central positions, showing no clear tendency toward either extreme of PC1 or PC2.

### 3.4. Microhabitat

PGLS analysis for the dorsal atlas surface revealed a significant size × microhabitat interaction, suggesting differences in allometric trajectories among microhabitat groups (R^2^ = 0.039; F = 1.881; Z = 1.834; *p* = 0.037). However, pairwise comparisons among groups were non-significant. Similarly, for the ventral atlas surface, the size × microhabitat interaction was significant (R^2^ = 0.043; F = 2.143; *p* = 0.034), and the interaction model fit the data better than the additive model (Z = 1.826; *p* = 0.034). Pairwise comparisons were again non-significant. Additional details are provided in Supplementary Material S2.

### 3.5. Locomotor Mode

PGLS analysis for the dorsal atlas surface detected no significant effect of locomotor mode or its interaction with size (R^2^ = 0.024; F = 1.077; *p* = 0.324). For the ventral surface, locomotor mode and its interaction with size were also non-significant (R^2^ = 0.043; F = 2.112; *p* = 0.060). Detailed results are provided in Supplementary Material S2.

## 4. Discussion

Although allometry was consistently significant, it explained only a relatively modest proportion of the atlas shape variations, particularly in the dorsal view. This indicates that body size, while representing an important axis of morphological diversification, is not sufficient to account for the full spectrum of atlas shape diversity observed across Hyloidea. The remaining variations likely reflect the combined influence of additional factors that were not explicitly examined in the present study, including aspects of feeding ecology, cranio-cervical biomechanics, developmental integration with adjacent skeletal structures, or lineage-specific functional specializations. These findings suggest that atlas shape diversification is likely driven by a multifactorial interplay of size, phylogenetic history, and additional ecological and biomechanical influences that remain to be explicitly tested.

Atlas morphology in hyloid anurans is structured primarily by allometric scaling and phylogenetic history. Morphological changes were concentrated mainly in atlas width and relative proportions, indicating that shape diversification occurs largely through size-dependent remodeling of this element. Although allometric trajectories suggested potential differences among ecological categories in some analyses, neither microhabitat nor locomotor mode independently accounted for substantial variations in atlas shape. In addition, the significant phylogenetic signal detected for both shape and size indicates that shared ancestry contributes to the distribution of atlas morphologies across lineages. Together, these findings suggest that atlas evolution in hyloid frogs occurs within a constrained structural framework in which scaling effects and phylogenetic history outweigh broad ecological partitioning, while still allowing clade-specific patterns of morphological divergence.

The allometric patterns observed differed between anatomical views, indicating non-uniform scaling of atlas morphology. In the dorsal view, shape variation was associated primarily with negative allometry (except in aquatic taxa), suggesting that some dorsal dimensions increase proportionally less than overall body size. In contrast, ventral morphology exhibited predominantly positive allometric trends, indicating relative expansion of ventral structures with increasing size. These contrasting patterns suggest that different anatomical regions of the atlas scale independently, potentially reflecting differential structural demands across the craniovertebral articulation. Within the context of a highly simplified cervical system, such allometric variations may represent compensatory remodeling of atlas shape in response to increasing biomechanical demands associated with body size.

Maher et al. [13] reported that amphibian necks are absolutely shorter and increase in length more slowly with body size than in other tetrapod groups, likely reflecting the strong structural constraint imposed by the presence of a single cervical vertebra. More broadly, across vertebrates, variations in neck length are commonly associated with ecologically driven changes in head size and function (e.g., [10,39,71,72]). Within this context, amphibians generally possess relatively large and broad heads relative to body size, a condition that likely imposes substantial mechanical constraints on the cervical region. In such cases, a shorter neck may enhance structural stability by reducing the mechanical leverage exerted by head mass on the cervical joints [6]. This condition reaches an extreme in anurans, where the cervical region is reduced to a single vertebra, the atlas, concentrating the mechanical demands of head support and mobility on this element alone.

Maher et al. [13] showed that neck length increases with body size to a lesser extent in carnivores than in herbivores, linking this pattern to the mechanical interplay between head size, feeding strategy, and cervical stability. In this context, the allometric patterns identified here may reflect biomechanical adjustments within a highly constrained cervical system, in which increasing functional demands are accommodated through shape modification rather than through elongation or addition of cervical elements. Consistent with this interpretation, some large-bodied predatory taxa within Hyloidea, such as species of *Ceratophrys* and *Lepidobatrachus*, with documented consumption of small vertebrates [73,74], exhibit particularly broadened atlases. Although this observation remains qualitative, it raises the possibility that feeding ecology may contribute to atlas shape variations in specific lineages and warrants explicit testing in future studies.

Our results show parallels with those reported by Bardua et al. [75] for cranial morphology, particularly in the strong influence of size on shape variation. As in the skull, atlas morphology exhibits a marked allometric component, with larger taxa displaying more robust configurations in which length and width approach similar proportions. In particular, smaller taxa (e.g., arboreal *Allophryne ruthveni*; Figure S1) tend to exhibit anteroposteriorly compressed atlases, whereas larger species (e.g., terrestrial *Leptodactylus laticeps*, Figure S1) display more robust morphologies, with length and width approaching more equivalent proportions. This pattern is broadly consistent with the “large size–wide skull” trend described for anurans [75], suggesting that size-related structural and functional constraints extend beyond the cranium to the cervical region. Furthermore, while Bardua et al. [75] report that aquatic taxa tend to exhibit larger skull sizes and occupy distinct regions of morphospace, our findings reveal a more nuanced pattern. Aquatic species are generally shifted toward larger atlas sizes, with most observations clustering at the upper end of the size distribution relative to other microhabitats (Supplementary Material S1, table with size data). Nevertheless, size ranges overlap across categories, and some non-aquatic taxa attain maximum values that exceed those observed in aquatic species. This pattern suggests a partial decoupling between size distribution and ecological categorization in the atlas.

In contrast to the skull, where microhabitat exerts a strong influence on morphology [75], ecological categories explained little independent variation in atlas shape. This suggests that, unlike the cranium, where feeding and sensory demands promote marked ecological differentiation [76,77,78,79], the atlas of hyloid anurans is more strongly constrained by structural and developmental factors. Rather than producing discrete morphological differentiation, ecological context may instead influence the way allometric shape change is expressed. In particular, the divergent allometric trajectories observed between aquatic and arboreal taxa, which occupy opposite ends of the locomotor and mechanical spectrum, may reflect contrasting functional demands related to hydrodynamic stabilization in aquatic environments versus fine control of head movements in complex three-dimensional substrates. In contrast, locomotor mode showed weaker associations with atlas shape variation, with no significant effects detected in PGLS models or most pairwise comparisons. Nevertheless, hoppers exhibited greater within-group shape variance than walkers in the ventral analysis, suggesting comparatively higher morphological dispersion within this locomotor category.

The significant phylogenetic signal detected in atlas morphology is largely driven (in dorsal view) by a small clade within the family Hylidae, formed by species of *Trachycephalus* and *Nyctimantis*, which cluster relatively close to one another in the phylomorphospace. (Figure 3; nodes 98–101). These taxa fall within the broader cluster of arboreal species but tend to occupy a similar region of morphospace with limited overlap with most other taxa. Notably, they correspond to relatively large-bodied frogs, particularly species of *Trachycephalus* (i.e., adult males 70–101 mm) [80], suggesting that their atlas morphology reflects the combined influence of phylogenetic affinity and body size. In these taxa, the atlas is markedly robust, with length and width approaching similar proportions, consistent with a structurally reinforced configuration that likely reflects the combined effects of shared ancestry and body size. This pattern highlights how clade-specific divergence can emerge within an otherwise constrained and broadly overlapping morphospace. An additional factor potentially contributing to the distinctive atlas morphology observed in *Trachycephalus* and *Nyctimantis* is the occurrence of phragmotic or semi-phragmotic behavior within some lophyohylin hylids [81,82]. In these taxa, individuals adopt a characteristic defensive posture in which the head is strongly flexed against the trunk and used to seal or partially obstruct refuge entrances [83]. Because this behavior relies on pronounced cranio-cervical flexion and precise control of head positioning, it may impose functional demands on the atlas and associated musculoskeletal structures beyond those associated with locomotion alone. Although this hypothesis remains speculative, it suggests that refuge-use behavior may represent an additional axis of functional differentiation shaping cervical morphology in specific arboreal lineages.

In a broader comparative context, a similar interplay between ecological factors and evolutionary history has been reported for cervical vertebrae in mammals [7] and birds [84], where variations in the shape of the atlas and axis has been linked to ecomorphological variables such as body size, locomotion, and possibly diet, while retaining a detectable phylogenetic component. However, the relative importance of these factors varies among clades: for example, atlas shape in primates has been associated with locomotor behavior [85], although phylogenetic history was not explicitly considered in those analyses, whereas studies in felids that incorporated phylogenetic information suggest a stronger influence of evolutionary history than of locomotion or diet [86]. Within this comparative framework, the significant phylogenetic signal observed in hyloid anurans indicates that evolutionary history contributes to atlas shape variations alongside allometric effects, further emphasizing the multifactorial nature of cervical morphological evolution across vertebrates.

Although ecological categories were not statistically significant, a suggestive trend emerges in morphospace: arboreal species (e.g., *Boana raniceps*, *Dendropsophus marmoratus*, *Nyctimantis brunoi*, *Trachycephalus mambaiensis*) cluster toward the right, whereas aquatic species shift toward the left (e.g., *Lepidobatrachus laevis*). Consistent with this pattern, arboreal taxa tend to exhibit a relatively narrower atlas, whereas aquatic species display comparatively wider ones. If biologically meaningful, this pattern may reflect subtle functional differentiation. A narrower atlas may permit greater dorsoventral or lateral head flexibility, facilitating fine head repositioning within complex three-dimensional arboreal substrates, a movement frequently observed in arboreal frogs, which actively bend and reorient the head while navigating vegetation (Figure 5). In contrast, a wider atlas may provide a broader articular base and improved distribution of mechanical loads generated by the head, a configuration that could be advantageous in aquatic environments where buoyancy and hydrodynamic forces act on both the body and the head. Within this framework, the steeper allometric trajectories observed in terrestrial and semi-aquatic species may be consistent with a greater degree of shape reorganization in response to increasing size-mechanical loads associated with head support and locomotion, whereas the comparatively weaker allometric patterns observed in arboreal taxa may suggest tighter constraints on atlas remodeling, potentially associated with the maintenance of cranial stability and precise head control in structurally complex environments. Alternatively, the relatively weak ecological differentiation observed in atlas morphology may reflect the functional versatility required by many anurans, which frequently exploit multiple substrates or environments throughout their life history. In this sense, the partial morphological uniformity of the atlas could represent a compromise associated with maintaining functional performance across distinct ecological contexts, as previously suggested for amphibians more broadly [87]. However, these interpretations remain speculative and require direct functional testing.

Experimental studies on head compensatory movements in anurans have failed to detect any association between microhabitat complexity or locomotor mode and the range of head movements, instead revealing a strong phylogenetic signal and suggesting that these movements may represent a conserved neuromotor reflex rather than an ecologically tuned performance trait [38]. Head compensatory movements constitute the primary mechanism of gaze stabilization in vertebrates and rely on tightly integrated vestibular and brainstem pathways [88]. The strong phylogenetic signal of this behavioral trait is consistent with the patterns observed here, suggesting that both morphology and performance may be shaped by shared evolutionary history, thereby weakening the expectation of a direct relationship with microhabitat. Notably, the greatest movement capacities have been reported in families such as Pyxicephalidae and Rhacophoridae [38], whereas the Hyloidea lineages examined in that study were restricted to only three families (i.e., Bufonidae, Hylidae and Dendrobatidae), representing a small fraction of the clade’s overall diversity. In this context, it becomes relevant to investigate how this trait has evolved within the highly diversified clade Hyloidea, especially given that head compensatory movements have been proposed as a basal condition in anurans [38]. Assessing its variation within this group, as well as its potential association with the morphology of head and neck structures, represents a promising avenue for future research.

In birds, shifts from ground foraging to arboreal gleaning have been linked to coordinated changes in cranial, appendicular, and axial morphology [84], functionally comparable to major habitat transitions such as the land-to-water shift [89,90]. A broadly analogous pattern might be expected in anurans, where transitions to arboreal microhabitats likely impose integrated functional demands across multiple anatomical systems. However, unlike birds, where the loss of forelimb grasping capacity has led to the hypothesis that the neck functions as a “surrogate forelimb” [10], arboreal anurans retain and extensively use their forelimbs for substrate interaction [91]. Consequently, the cervical region in anurans may experience weaker selective pressure for functional diversification, potentially explaining why the atlas remains structurally conserved as a single vertebral element despite subtle variations in shape.

Overall, our findings indicate that atlas shape evolution in Hyloidea reflects the combined influence of allometric scaling, phylogenetic history, and ecological context within a structurally simplified cervical system. Future studies integrating atlas morphology with cranial morphology and other components of the cranio-cervical system, such as the hyoid apparatus, may provide a more comprehensive understanding of the evolutionary integration of these structures. Likewise, incorporating data on feeding ecology, locomotor performance, and cranio-cervical biomechanics will help clarify the functional significance of the morphological patterns identified here and determine how variations in atlas shape contribute to head mobility, ecological specialization, and the evolution of the anuran feeding and locomotor apparatus.

## 5. Conclusions

Despite the extreme structural simplification of the anuran cervical region, the atlas exhibits appreciable morphological variations across hyloid frogs. However, these variations are structured primarily by allometric scaling and phylogenetic history rather than by discrete ecological categories such as microhabitat or locomotor mode. These findings indicate that atlas evolution in hyloid anurans occurs within a constrained structural framework in which morphological diversification is achieved mainly through size-dependent remodeling rather than ecological specialization. More broadly, our results suggest that an extreme reduction in the cervical system channels morphological evolution along restricted allometric and phylogenetic pathways, limiting the extent to which ecological divergence can shape cervical anatomy.

## Figures and Tables

**Table 1:** Description of dorsal and ventral atlas landmarks (LM) used in geometric morphometric analyses.

LM	Dorsal	LM	Ventral
1	Anterior midpoint of the neural arch.	1	Anterior midpoint of the vertebral centra.
2, 3	Junction between the neural arch and the atlantal cotyle.	2, 3	Medial extreme point of the atlantal cotyle.
4, 5	Lateral extreme point of the atlantal cotyle.	4, 5	Lateral extreme point of the atlantal cotyle.
6, 7	Inflection point on the junction between the cotyle and postzygapophyses.	6, 7	Lateral extreme point of the vertebral centra.
8, 9	Posterior extreme point of the postzygapophyses.	8	Posterior midpoint of the vertebral centra.
10, 11	Medial point of the postzygapophyses.		
12	Posterior midpoint of the neural arch.		

## Data Availability

The data supporting the findings of this study are available in the Supplementary Materials accompanying this article.

## Supplementary Materials

The following supporting information can be downloaded at: https://www.mdpi.com/article/10.3390/biology15141200/s1, refs. [92,93,94,95,96,97,98,99,100,101,102,103,104,105,106,107,108,109,110,111,112,113,114,115,116,117,118,119,120,121,122,123,124,125,126,127,128,129,130,131,132,133] are cited in SM file.
