# Supplementary material for: Constraint and Allometric Diversification in a Simplified Neck: Shape Evolution of the Atlas in Hyloidea (Anura)
Source: Biology (Basel). 2026 Jul 20;15(14):1200. doi: 10.3390/biology15141200 (PMC13405607; doi:10.3390/biology15141200)
Supplement: Supplementary file 1 [file biology-15-01200-s001.zip › Fig S1.pdf]

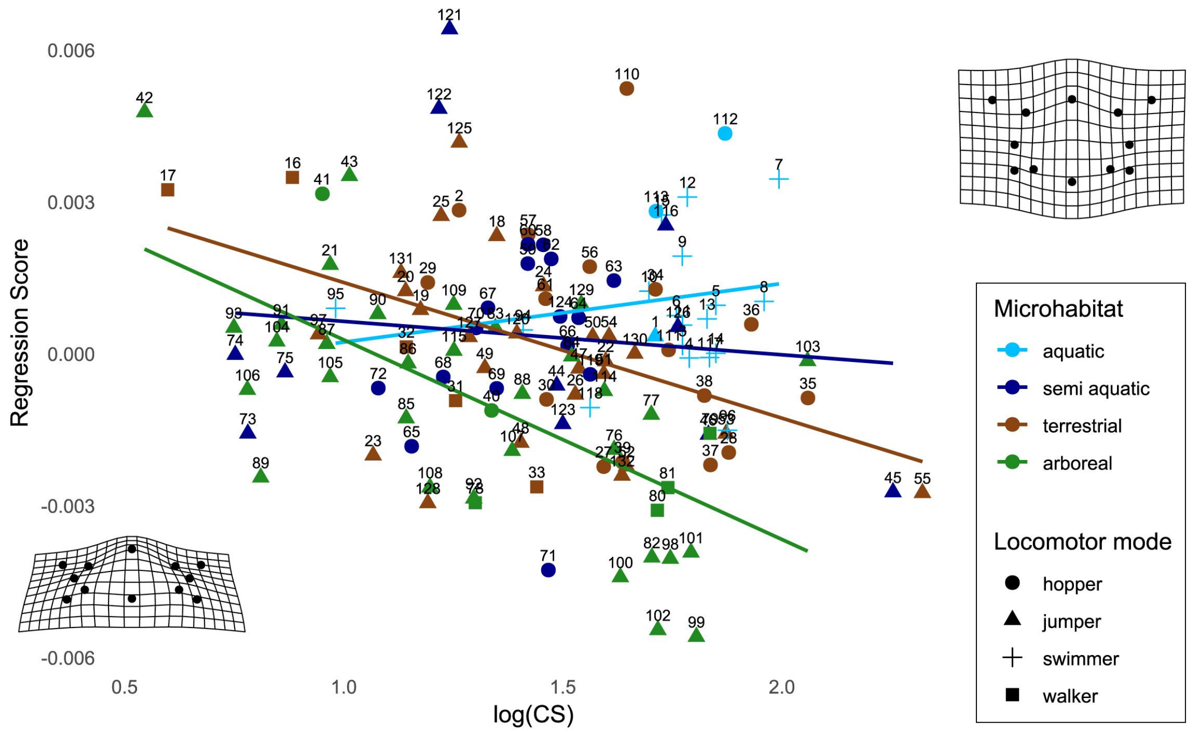

Figure S1. Allometric trajectories of the atlas in dorsal view. The X-axis represents the natural logarithm of centroid size (log CS) as a proxy for body size, and the Y-axis represents the Regression Score (RegScore method), a univariate score summarizing size-associated shape variation. Colors represent habitat categories: aquatic (blue), semi-aquatic (dark blue), terrestrial (brown), and arboreal (green). Symbols represent locomotor mode categories: circle (hopper), triangle (jumper), plus sign (swimmer), and square (walker). Regression lines represent the allometric trajectory of each microhabitat category. Deformation grids represent the mean shape of the minimum (left) and maximum (right) predicted shapes for each atlas view. Numbers correspond to species as follows: (1) *Insuetophrynus acarpicus*, (2) *Rhinoderma darwinii*, (3) *Telmatobius ceiorum*, (4) *Telmatobius stephani*, (5) *Telmatobius rubigo*, (6) *Telmatobius atacamensis*, (7) *Telmatobius hypselocephalus*, (8) *Telmatobius platycephalus*, (9) *Telmatobius contrerasi*, (10) *Telmatobius hauthali*, (11) *Telmatobius oxycephalus*, (12) *Telmatobius pinguiculus*, (13) *Telmatobius pisanoi*, (14) *Telmatobius schreiteri*, (15) *Telmatobius scrocchii*, (16) *Brachycephalus* aff. *margaritatus*, (17) *Brachycephalus darkside*, (18) *Oreobates discoidalis*, (19) *Oreobates berdemenos*, (20) *Eleutherodactylus rufescens*, (21) *Ceuthomantis smaragdinus*, (22) *Phyllobates bicolor*, (23) *Oophaga pumilio*, (24) *Hyloxalus fuliginosus*, (25) *Ameerega picta*, (26) *Ameerega trivittata*, (27) *Odontophrynus americanus*, (28) *Odontophrynus asper*, (29) *Proceratophrys avelinoi*, (30) *Proceratophrys melanopogon*, (31) *Melanophryniscus tumifrons*, (32) *Melanophryniscus klappenbachi*, (33) *Melanophryniscus rubriventris*, (34) *Rhinella achalensis*, (35) *Rhinella*

spinulosa, (36) *Rhinella arenarum*, (37) *Rhinella crucifer* x *ornata*, (38) *Rhinella dorbignyi*, (39) *Rhinella major*, (40) *Dendrophryniscus proboscideus*, (41) *Dendrophryniscus brevipollicatus*, (42) *Allophryne ruthveni*, (43) *Vitreorana parvula*, (44) *Leptodactylus podicipinus*, (45) *Leptodactylus insularum*, (46) *Leptodactylus macrosternum*, (47) *Leptodactylus fuscus*, (48) *Leptodactylus fragilis*, (49) *Leptodactylus latinasus*, (50) *Leptodactylus gracilis*, (51) *Leptodactylus plaumanni*, (52) *Leptodactylus elenae*, (53) *Leptodactylus mystacinus*, (54) *Leptodactylus bufonius*, (55) *Leptodactylus laticeps*, (56) *Pleurodema thaul*, (57) *Pleurodema kriegi*, (58) *Pleurodema cordobae*, (59) *Pleurodema bibroni*, (60) *Pleurodema guayapae*, (61) *Pleurodema nebulosum*, (62) *Pleurodema tucumanum*, (63) *Pleurodema borellii*, (64) *Pleurodema cinereum*, (65) *Engystomops pustulosus*, (66) *Physalaemus nattereri*, (67) *Physalaemus cuvieri*, (68) *Physalaemus albonotatus*, (69) *Physalaemus biligonigerus*, (70) *Physalaemus santafecinus*, (71) *Physalaemus maximus*, (72) *Physalaemus feioi*, (73) *Pseudopaludicola mystacalis*, (74) *Pseudopaludicola boliviana*, (75) *Pseudopaludicola falcipes*, (76) *Agalychnis callidryas*, (77) *Agalychnis moreletii*, (78) *Pithecopus azureus*, (79) *Phyllomedusa boliviana*, (80) *Phyllomedusa tetraploidea*, (81) *Phyllomedusa sauvagii*, (82) *Boana raniceps*, (83) *Boana riojana*, (84) *Boana cordobae*, (85) *Boana pulchella*, (86) *Boana curupi*, (87) *Boana semiguttata*, (88) *Aplastodiscus perviridis*, (89) *Dendropsophus nanus*, (90) *Dendropsophus elegans*, (91) *Dendropsophus minutus*, (92) *Dendropsophus marmoratus*, (93) *Dendropsophus decipiens*, (94) *Pseudis minuta*, (95) *Pseudis limellum*, (96) *Pseudis platensis*, (97) *Acris crepitans*, (98) *Trachycephalus typhonius*, (99) *Trachycephalus mambaiensis*, (100) *Trachycephalus atlas*, (101) *Nyctimantis siemersi*, (102) *Nyctimantis brunoi*, (103) *Itapotihyla langsdorffii*, (104) *Ololygon berthae*, (105) *Ololygon carnevallii*, (106) *Scinax fuscomarginatus*, (107) *Scinax fuscovarius*, (108) *Scinax nasicus*, (109) *Scinax acuminatus*, (110) *Ceratophrys cranwelli*, (111) *Chacophrys pierottii*, (112) *Lepidobatrachus laevis*, (113) *Lepidobatrachus llanensis*, (114) *Gastrotheca chrysosticta*, (115) *Gastrotheca* cf. *christiani*, (116) *Limnomedusa macroglossa*, (117) *Alsodes gargola*, (118) *Alsodes neuquensis*, (119) *Eupsophus roseus*, (120) *Batrachyla taeniata*, (121) *Atelognathus patagonicus*, (122) *Atelognathus nitoi*, (123) *Atelognathus reverberii*, (124) *Chaltenobatrachus grandisonae*, (125) *Batrachyla leptopus*, (126) *Hylorina sylvatica*, (127) *Crossodactylus schmidti*, (128) *Crossodactylus gaudichaudii*, (129) *Hylodes nasus*, (130) *Thoropa miliaris*, (131) *Thoropa bryomantis*, (132) *Cycloramphus boraceiensis*.
