# Supplementary material for: Constraint and Allometric Diversification in a Simplified Neck: Shape Evolution of the Atlas in Hyloidea (Anura)
Source: Biology (Basel). 2026 Jul 20;15(14):1200. doi: 10.3390/biology15141200 (PMC13405607; doi:10.3390/biology15141200)
Supplement: Supplementary file 1 [file biology-15-01200-s001.zip › Fig S3.pdf]

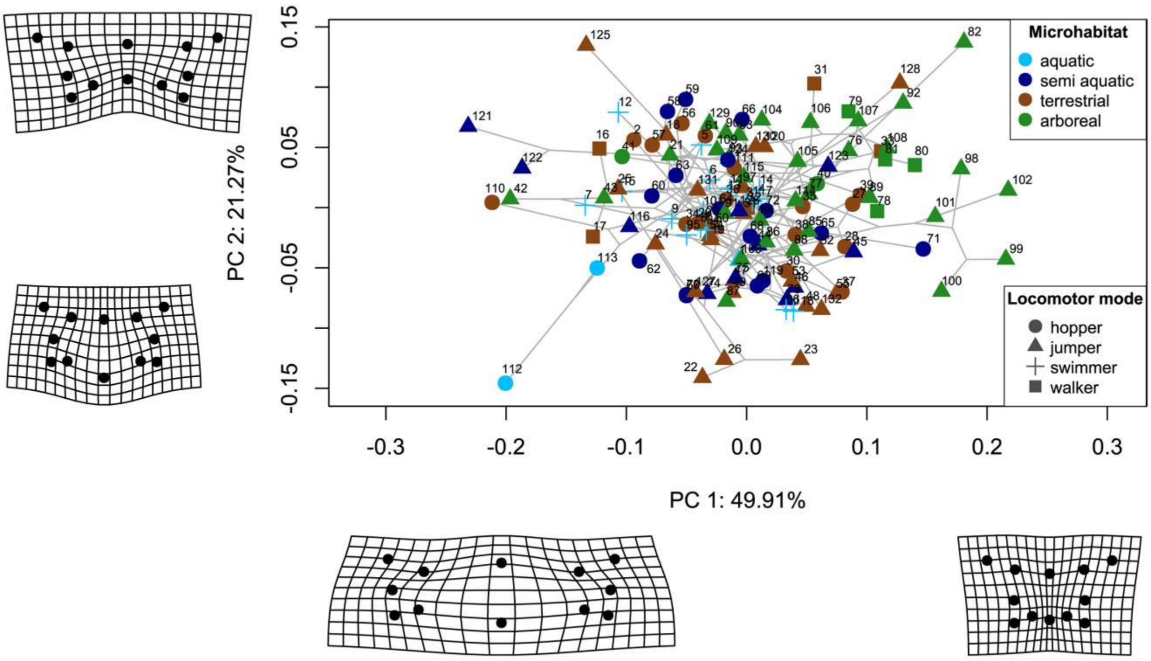

Figure S3. Phylogenetic principal component analysis (pPCA) of atlas shape in dorsal view based on geometric morphometric data. The first two principal components explain 49.91% (PC1) and 21.27% (PC2) of the total shape variation. Deformation grids illustrate the shape changes associated with the minimum and maximum extremes of PC1 and PC2. Each point represents a species, numbered according to the list in the caption, and connected by thin grey lines to the phylogenetic structure. Colors represent habitat categories: aquatic (blue), semi-aquatic (dark blue), terrestrial (brown), and arboreal (green). Symbols represent locomotor mode categories: circle (hopper), triangle (jumper), plus sign (swimmer), and square (walker). Numbers correspond to species as follows: (1) *Insuetophrynus acarpicus*, (2) *Rhinoderma darwinii*, (3) *Telmatobius ceiorum*, (4) *Telmatobius stephani*, (5) *Telmatobius rubigo*, (6) *Telmatobius atacamensis*, (7) *Telmatobius hypselocephalus*, (8) *Telmatobius platycephalus*, (9) *Telmatobius contrerasi*, (10) *Telmatobius hauthali*, (11) *Telmatobius oxycephalus*, (12) *Telmatobius pinguiculus*, (13) *Telmatobius pisanoi*, (14) *Telmatobius schreiteri*, (15) *Telmatobius scrocchii*, (16) *Brachycephalus aff. margaritatus*, (17) *Brachycephalus darkside*, (18) *Oreobates discoidalis*, (19) *Oreobates berdemenos*, (20) *Eleutherodactylus rufescens*, (21) *Ceuthomantis smaragdinus*, (22) *Phyllobates bicolor*, (23) *Oophaga pumilio*, (24) *Hyloxalus fuliginosus*, (25) *Ameerega picta*, (26) *Ameerega trivittata*, (27) *Odontophrynus americanus*, (28) *Odontophrynus asper*, (29) *Proceratophrys avelinoi*, (30) *Proceratophrys melanopogon*, (31) *Melanophryniscus tumifrons*, (32) *Melanophryniscus klappenbachi*, (33) *Melanophryniscus rubriventris*, (34) *Rhinella achalensis*, (35) *Rhinella spinulosa*, (36)

Rhinella arenarum, (37) *Rhinella crucifer* × *ornata*, (38) *Rhinella dorbignyi*, (39) *Rhinella major*, (40) *Dendrophryniscus proboscideus*, (41) *Dendrophryniscus brevipollicatus*, (42) *Allophryne ruthveni*, (43) *Vitreorana parvula*, (44) *Leptodactylus podicipinus*, (45) *Leptodactylus insularum*, (46) *Leptodactylus macrosternum*, (47) *Leptodactylus fuscus*, (48) *Leptodactylus fragilis*, (49) *Leptodactylus latinasus*, (50) *Leptodactylus gracilis*, (51) *Leptodactylus plaumanni*, (52) *Leptodactylus elenae*, (53) *Leptodactylus mystacinus*, (54) *Leptodactylus bufonius*, (55) *Leptodactylus laticeps*, (56) *Pleurodema thaul*, (57) *Pleurodema kriegi*, (58) *Pleurodema cordobae*, (59) *Pleurodema bibroni*, (60) *Pleurodema guayapae*, (61) *Pleurodema nebulosum*, (62) *Pleurodema tucumanum*, (63) *Pleurodema borellii*, (64) *Pleurodema cinereum*, (65) *Engystomops pustulosus*, (66) *Physalaemus nattereri*, (67) *Physalaemus cuvieri*, (68) *Physalaemus albonotatus*, (69) *Physalaemus biligonigerus*, (70) *Physalaemus santafecinus*, (71) *Physalaemus maximus*, (72) *Physalaemus feioi*, (73) *Pseudopaludicola mystacalis*, (74) *Pseudopaludicola boliviana*, (75) *Pseudopaludicola falcipes*, (76) *Agalychnis callidryas*, (77) *Agalychnis moreletii*, (78) *Pithecopus azureus*, (79) *Phyllomedusa boliviana*, (80) *Phyllomedusa tetraploidea*, (81) *Phyllomedusa sauvagii*, (82) *Boana raniceps*, (83) *Boana riojana*, (84) *Boana cordobae*, (85) *Boana pulchella*, (86) *Boana curupi*, (87) *Boana semiguttata*, (88) *Aplastodiscus perviridis*, (89) *Dendropsophus nanus*, (90) *Dendropsophus elegans*, (91) *Dendropsophus minutus*, (92) *Dendropsophus marmoratus*, (93) *Dendropsophus decipiens*, (94) *Pseudis minuta*, (95) *Pseudis limellum*, (96) *Pseudis platensis*, (97) *Acris crepitans*, (98) *Trachycephalus typhonius*, (99) *Trachycephalus mambaiensis*, (100) *Trachycephalus atlas*, (101) *Nyctimantis siemersi*, (102) *Nyctimantis brunoi*, (103) *Itapotihyla langsdorffii*, (104) *Ololygon berthae*, (105) *Ololygon carnevallii*, (106) *Scinax fuscomarginatus*, (107) *Scinax fuscovarius*, (108) *Scinax nasicus*, (109) *Scinax acuminatus*, (110) *Ceratophrys cranwelli*, (111) *Chacophrys pierottii*, (112) *Lepidobatrachus laevis*, (113) *Lepidobatrachus llanensis*, (114) *Gastrotheca chrysosticta*, (115) *Gastrotheca cf. christiani*, (116) *Limnomedusa macroglossa*, (117) *Alsodes gargola*, (118) *Alsodes neuquensis*, (119) *Eupsophus roseus*, (120) *Batrachyla taeniata*, (121) *Atelognathus patagonicus*, (122) *Atelognathus nitoi*, (123) *Atelognathus reverberii*, (124) *Chaltenobatrachus grandisonae*, (125) *Batrachyla leptopus*, (126) *Hylorina sylvatica*, (127) *Crossodactylus schmidti*, (128) *Crossodactylus gaudichaudii*, (129) *Hylodes nasus*, (130) *Thoropa miliaris*, (131) *Thoropa bryomantis*, and (132) *Cycloramphus boraceiensis*.
