# Supplementary material for: Constraint and Allometric Diversification in a Simplified Neck: Shape Evolution of the Atlas in Hyloidea (Anura)
Source: Biology (Basel). 2026 Jul 20;15(14):1200. doi: 10.3390/biology15141200 (PMC13405607; doi:10.3390/biology15141200)
Supplement: Supplementary file 1 [file biology-15-01200-s001.zip › Fig S4.pdf]

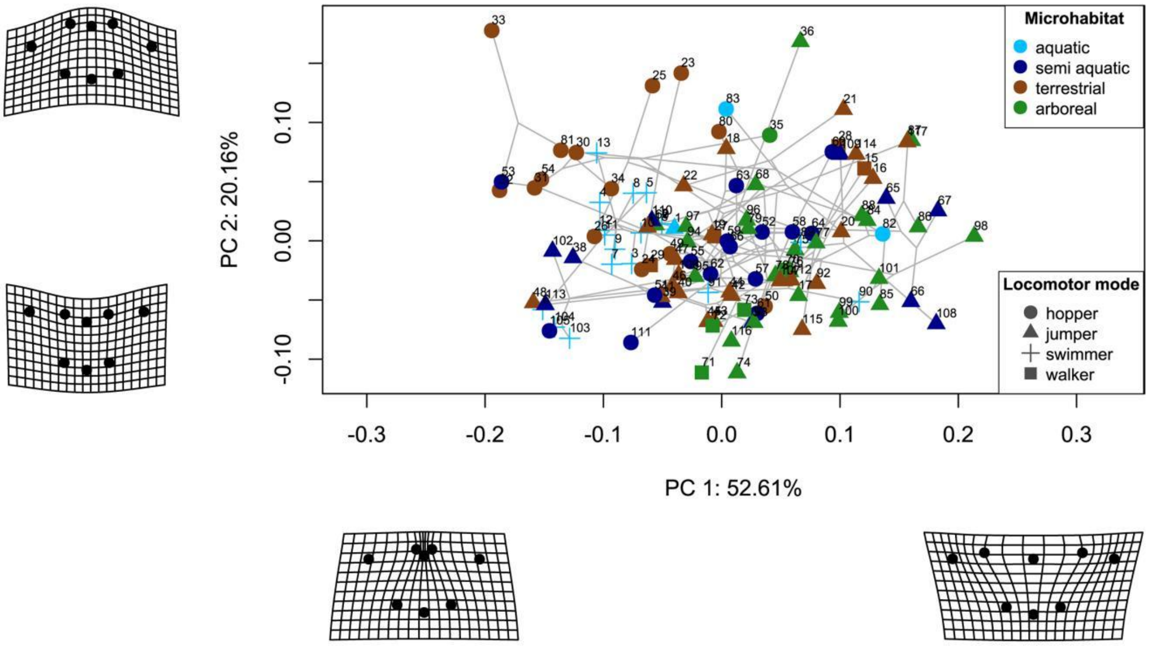

Figure S4. Phylogenetic principal component analysis (pPCA) of atlas shape in ventral view based on geometric morphometric data. The first two principal components explain 52.61% (PC1) and 20.16% (PC2) of the total shape variation. Deformation grids illustrate the shape changes associated with the minimum and maximum extremes of PC1 and PC2. Each point represents a species, numbered according to the list in the caption, and connected by thin grey lines to the phylogenetic structure. Colors represent habitat categories: aquatic (blue), semi-aquatic (dark blue), terrestrial (brown), and arboreal (green). Symbols represent locomotor mode categories: circle (hopper), triangle (jumper), plus sign (swimmer), and square (walker). Numbers correspond to species as follows: (1) *Insuetophrynus acarpicus*, (2) *Telmatobius ceiorum*, (3) *Telmatobius stephani*, (4) *Telmatobius rubigo*, (5) *Telmatobius atacamensis*, (6) *Telmatobius platycephalus*, (7) *Telmatobius contrerasi*, (8) *Telmatobius hauthali*, (9) *Telmatobius oxycephalus*, (10) *Telmatobius pinguiculus*, (11) *Telmatobius pisanoi*, (12) *Telmatobius schreiteri*, (13) *Telmatobius scrocchii*, (14) *Oreobates discoidalis*, (15) *Brachycephalus* aff. *margaritatus*, (16) *Eleutherodactylus rufescens*, (17) *Ceuthomantis smaragdinus*, (18) *Phyllobates bicolor*, (19) *Oophaga pumilio*, (20) *Hyloxalus fuliginosus*, (21) *Ameerega picta*, (22) *Ameerega trivittata*, (23) *Odontophrynus americanus*, (24) *Odontophrynus asper*, (25) *Proceratophrys avelinoi*, (26) *Proceratophrys melanopogon*, (27) *Melanophryniscus tumifrons*, (28) *Melanophryniscus klappenbachi*, (29) *Melanophryniscus rubriventris*, (30) *Rhinella achalensis*, (31) *Rhinella spinulosa*, (32) *Rhinella arenarum*, (33) *Rhinella crucifer* × *ornata*, (34) *Rhinella major*, (35) *Dendrophryniscus brevipollicatus*, (36)

Allophryne ruthveni, (37) *Leptodactylus podicipinus*, (38) *Leptodactylus insularum*, (39) *Leptodactylus macrosternum*, (40) *Leptodactylus fuscus*, (41) *Leptodactylus fragilis*, (42) *Leptodactylus latinasus*, (43) *Leptodactylus gracilis*, (44) *Leptodactylus plaumanni*, (45) *Leptodactylus elenae*, (46) *Leptodactylus mystacinus*, (47) *Leptodactylus bufonius*, (48) *Leptodactylus laticeps*, (49) *Pleurodema thaul*, (50) *Pleurodema kriegi*, (51) *Pleurodema cordobae*, (52) *Pleurodema bibroni*, (53) *Pleurodema guayapae*, (54) *Pleurodema nebulosum*, (55) *Pleurodema tucumanum*, (56) *Pleurodema borellii*, (57) *Pleurodema cinereum*, (58) *Engystomops pustulosus*, (59) *Physalaemus nattereri*, (60) *Physalaemus cuvieri*, (61) *Physalaemus albonotatus*, (62) *Physalaemus biligonigerus*, (63) *Physalaemus santafecinus*, (64) *Physalaemus feioi*, (65) *Pseudopaludicola mystacalis*, (66) *Pseudopaludicola boliviana*, (67) *Pseudopaludicola falcipes*, (68) *Agalychnis callidryas*, (69) *Agalychnis moreletii*, (70) *Pithecopus azureus*, (71) *Phyllomedusa boliviana*, (72) *Phyllomedusa tetraploidea*, (73) *Phyllomedusa sauvagii*, (74) *Boana raniceps*, (75) *Boana riojana*, (76) *Boana cordobae*, (77) *Boana pulchella*, (78) *Boana curupi*, (79) *Aplastodiscus perviridis*, (80) *Ceratophrys cranwelli*, (81) *Chacophrys pierottii*, (82) *Lepidobatrachus laevis*, (83) *Lepidobatrachus llanensis*, (84) *Gastrotheca chrysosticta*, (85) *Dendropsophus nanus*, (86) *Dendropsophus elegans*, (87) *Dendropsophus minutus*, (88) *Dendropsophus marmoratus*, (89) *Pseudis minuta*, (90) *Pseudis limellum*, (91) *Pseudis platensis*, (92) *Acris crepitans*, (93) *Trachycephalus typhonius*, (94) *Trachycephalus mambaiensis*, (95) *Nyctimantis siemersi*, (96) *Nyctimantis brunoi*, (97) *Itapotihyla langsdorffii*, (98) *Ololygon berthae*, (99) *Scinax fuscovarius*, (100) *Scinax nasicus*, (101) *Scinax acuminatus*, (102) *Limnomedusa macroglossa*, (103) *Alsodes gargola*, (104) *Alsodes neuquensis*, (105) *Eupsophus roseus*, (106) *Batrachyla taeniata*, (107) *Batrachyla antartandica*, (108) *Atelognathus patagonicus*, (109) *Atelognathus nitoi*, (110) *Atelognathus reverberii*, (111) *Chaltenobatrachus grandisonae*, (112) *Batrachyla leptopus*, (113) *Hylorina sylvatica*, (114) *Crossodactylus schmidti*, (115) *Crossodactylus gaudichaudii*, (116) *Hylodes nasus*, (117) *Thoropa bryomantis*, (118) *Thoropa miliaris*.
