# Supplementary material for: Constraint and Allometric Diversification in a Simplified Neck: Shape Evolution of the Atlas in Hyloidea (Anura)
Source: Biology (Basel). 2026 Jul 20;15(14):1200. doi: 10.3390/biology15141200 (PMC13405607; doi:10.3390/biology15141200)
Supplement: Supplementary file 1 [file biology-15-01200-s001.zip › Supplementary Material S2.pdf]

Supplementary Material S2

Results of the geometric morphometric analyses of atlas shape in dorsal and ventral views. The supplementary tables include analyses of bilateral symmetry, static and evolutionary allometry, phylogenetic signal, phylogenetic generalized least-squares (PGLS) models evaluating the effects of microhabitat and locomotor mode on atlas shape, and pairwise comparisons among ecological categories.

Dorsal view

Table S2.1 – Bilateral symmetry

Results of bilateral symmetry analysis (object symmetry, procD.lm with RRPP) for atlas shape in dorsal view. The symmetric component of shape was retained for all subsequent analyses.

Source	Df	SS	MS	R^2	F	Z	p
Individuals	444	16.4996	0.037161	0.92484	13.047	30.1451	0.001 ***
Side	1	0.0762	0.076214	0.00427	26.759	6.6059	0.001 ***
Individuals × Side	444	1.2646	0.002848	0.07088	—	—	—
Total	889	17.8404	—	—	—	—	—

*Significance codes: 0 '***' 0.001 '**' 0.01 '*' 0.05 '.' 0.1 ' ' 1. 1000 permutations (RRPP).*

Table S2.2 – Static allometry (OLS)

Multivariate regression of atlas shape on log-transformed centroid size (log CS) estimated by Ordinary Least Squares (OLS), without phylogenetic correction. Dorsal view; n = 132 species.

Source	Df	SS	MS	R^2	F	Z	p
log(CS)	1	0.06143	0.061433	0.03326	4.4727	2.3544	0.006 **
Residuals	130	1.78556	0.013735	0.96674	—	—	—
Total	131	1.84699	—	—	—	—	—

*Significance codes: 0 '***' 0.001 '**' 0.01 '*' 0.05 '.' 0.1 ' ' 1. 1000 permutations (RRPP).*

Table S2.3 – Evolutionary allometry (PGLS)

Multivariate regression of atlas shape on log CS estimated by Phylogenetic Generalized Least Squares (PGLS), incorporating phylogenetic covariance. Dorsal view; n = 132 species.

Source	Df	SS	MS	R^2	F	Z	p
log(CS)	1	3.820	3.8202	0.06034	8.3475	3.6425	0.001 ***
Residuals	130	59.494	0.4576	0.93966	—	—	—
Total	131	63.314	—	—	—	—	—

*Significance codes: 0 '***' 0.001 '**' 0.01 '*' 0.05 '.' 0.1 ' ' 1. 1000 permutations (RRPP).*

Table S2.4 – Phylogenetic signal

Phylogenetic signal estimated for atlas shape (multivariate; physignal.z, λ optimized via 'burn' method) and centroid size (univariate; physignal, Blomberg's K) in dorsal view. Results based on 1000 permutations.

Variable	Statistic	Value	p
Shape (Procrustes coordinates)	Z (effect size)	7.0898	0.001
Shape (Procrustes coordinates)	λ (Pagel's lambda)	1	—
Shape (Procrustes coordinates)	K (Brownian Motion ratio)	0.04	—
Centroid size	K (Blomberg's K)	0.0576	0.001

*Significance codes: 0 '***' 0.001 '**' 0.01 '*' 0.05 '.' 0.1 ' ' 1. 1000 permutations (RRPP).*

Table S2.5 – Effect of microhabitat on atlas shape (additive PGLS model)

PGLS model testing the additive effects of log CS and microhabitat on atlas shape in dorsal view. Four microhabitat categories: aquatic, arboreal, semi-aquatic, terrestrial. n = 132 species.

Source	Df	SS	MS	R^2	F	Z	p
log(CS)	1	3.820	3.8202	0.06034	8.4836	3.6742	0.001 ***
Microhabitat	3	2.305	0.7683	0.03640	1.7061	1.4709	0.065 .
Residuals	127	57.189	0.4503	0.90326	—	—	—
Total	131	63.314	—	—	—	—	—

*Significance codes: 0 '***' 0.001 '**' 0.01 '*' 0.05 '.' 0.1 ' ' 1. 1000 permutations (RRPP).*

Table S2.6 – Heterogeneous allometry by microhabitat (interaction PGLS model)

PGLS model testing the interaction between log CS and microhabitat, evaluating whether allometric trajectories differ among microhabitat categories. Dorsal view; n = 132 species.

Source	Df	SS	MS	R^2	F	Z	p
log(CS)	1	3.820	3.8202	0.06034	8.6380	3.6884	0.001 ***
Microhabitat	3	2.158	0.7192	0.03408	1.6263	1.3496	0.099 .
log(CS) × Microhabitat	3	2.496	0.8320	0.03942	1.8813	1.8340	0.037 *
Residuals	124	54.840	0.4423	0.86616	—	—	—
Total	131	63.314	—	—	—	—	—

*Significance codes: 0 '***' 0.001 '**' 0.01 '*' 0.05 '.' 0.1 ' ' 1. 1000 permutations (RRPP).*

Table S2.7 – Nested model comparison: microhabitat

Nested ANOVA comparing the additive model (shape ~ log CS + microhabitat) and the interaction model (shape ~ log CS × microhabitat). Dorsal view.

Model	Res. Df	Df	RSS	SS	MS	R^2	F	Z	p
shape ~ log(CS) + Microhabitat	127	1	57.336	—	—	0.000000	—	—	—
shape ~ log(CS) × Microhabitat	124	3	54.840	2.4961	0.83204	0.039424	1.8813	1.834	0.037

*Significance codes: 0 '***' 0.001 '**' 0.01 '*' 0.05 '.' 0.1 ' ' 1. 1000 permutations (RRPP).*

Table S2.8 – Pairwise comparisons among microhabitat categories

All pairwise tests performed using the interaction model as full model and the additive model as null model, with 1000 RRPP permutations (95% confidence intervals). Dorsal view.

(a) Euclidean distances between group means in shape space (dist)

Comparison	d	UCL (95%)	Z	p
Aquatic – Arboreal	0.12187	0.20352	−0.6509	0.756
Aquatic – Semi-aquatic	0.04683	0.11670	−1.4714	0.928
Aquatic – Terrestrial	0.08225	0.14295	−0.9757	0.839
Arboreal – Semi-aquatic	0.08577	0.16142	−0.5875	0.735
Arboreal – Terrestrial	0.05480	0.12715	−0.9210	0.815
Semi-aquatic – Terrestrial	0.04443	0.07911	−0.6949	0.762

*Significance codes: 0 '***' 0.001 '**' 0.01 '*' 0.05 '.' 0.1 ' ' 1. 1000 permutations (RRPP).*

(b) Correlations between allometric vectors (VC)

Comparison	r	Angle	UCL (95%)	Z	p
Aquatic – Arboreal	0.9926	0.12164	0.19169	−0.3662	0.653
Aquatic – Semi-aquatic	0.9989	0.04676	0.10923	−1.2520	0.898
Aquatic – Terrestrial	0.9966	0.08215	0.13314	−0.6845	0.754
Arboreal – Semi-aquatic	0.9963	0.08560	0.15046	−0.3547	0.649
Arboreal – Terrestrial	0.9985	0.05465	0.11835	−0.7118	0.755
Semi-aquatic – Terrestrial	0.9990	0.04439	0.07380	−0.4440	0.678

*Significance codes: 0 '***' 0.001 '**' 0.01 '*' 0.05 '.' 0.1 ' ' 1. 1000 permutations (RRPP).*

(c) Differences in allometric vector lengths (DL)

Comparison	d	UCL (95%)	Z	p
Aquatic – Arboreal	0.001234	0.044118	−1.6525	0.949
Aquatic – Semi-aquatic	0.000831	0.026374	−1.5550	0.927
Aquatic – Terrestrial	0.000936	0.032764	−1.6935	0.945
Arboreal – Semi-aquatic	0.002065	0.031970	−1.1611	0.855
Arboreal – Terrestrial	0.002170	0.025012	−0.8716	0.797
Semi-aquatic – Trimestral	0.000105	0.016072	−2.0972	0.983

*Significance codes: 0 '***' 0.001 '**' 0.01 '*' 0.05 '.' 0.1 ' ' 1. 1000 permutations (RRPP).*

(d) Within-group shape variances (var)

Observed variances by group — Aquatic: 0.03801; Arboreal: 0.03722; Semi-aquatic: 0.03703; Terrestrial: 0.03630.

Comparison	d	UCL (95%)	Z	p
Aquatic – Arboreal	0.000795	0.003335	−0.2972	0.606
Aquatic – Semi-aquatic	0.000989	0.002277	0.4371	0.347
Aquatic – Terrestrial	0.001710	0.002149	1.2194	0.112
Arboreal – Semi-aquatic	0.000194	0.003134	−1.3254	0.899
Arboreal – Terrestrial	0.000915	0.003333	−0.0351	0.516
Semi-aquatic – Terrestrial	0.000721	0.001588	0.2957	0.408

*Significance codes: 0 '***' 0.001 '**' 0.01 '*' 0.05 '.' 0.1 ' ' 1. 1000 permutations (RRPP).*

Table S2.9 – Effect of locomotor mode on atlas shape (additive PGLS model)

PGLS model testing the additive effects of log CS and locomotor mode on atlas shape in dorsal view. Four locomotor categories: hopper, jumper, swimmer, walker. n = 132 species.

Source	Df	SS	MS	R^2	F	Z	p
log(CS)	1	3.820	3.8202	0.06034	8.2441	3.6192	0.001 ***
Locomotor mode	3	0.644	0.2146	0.01017	0.4632	−1.4013	0.925
Residuals	127	58.850	0.4634	0.92949	—	—	—
Total	131	63.314	—	—	—	—	—

*Significance codes: 0 '***' 0.001 '**' 0.01 '*' 0.05 '.' 0.1 ' ' 1. 1000 permutations (RRPP).*

Table S2.10 – Heterogeneous allometry by locomotor mode (interaction PGLS model)

PGLS model testing the interaction between log CS and locomotor mode. Dorsal view; n = 132 species.

Source	Df	SS	MS	R^2	F	Z	p
log(CS)	1	3.820	3.8202	0.06034	8.2592	3.6058	0.001 ***
Locomotor mode	3	0.644	0.2146	0.01017	0.4640	−1.3954	0.922
log(CS) × Locomotor mode	3	1.495	0.4983	0.02361	1.0774	0.4274	0.324
Residuals	124	57.355	0.4625	0.90588	—	—	—
Total	131	63.314	—	—	—	—	—

*Significance codes: 0 '***' 0.001 '**' 0.01 '*' 0.05 '.' 0.1 ' ' 1. 1000 permutations (RRPP).*

Table S2.11 – Nested model comparison: locomotor mode

Nested ANOVA comparing the additive model (shape ~ log CS + locomotor mode) and the interaction model (shape ~ log CS × locomotor mode). Dorsal view.

Model	Res. Df	Df	RSS	SS	MS	R^2	F	Z	p
shape ~ log(CS) + Locomotor mode	127	1	58.850	—	—	0.000000	—	—	—
shape ~ log(CS) × Locomotor mode	124	3	57.355	1.495	0.49835	0.023613	1.0774	0.4274	0.324

*Significance codes: 0 '***' 0.001 '**' 0.01 '*' 0.05 '.' 0.1 ' ' 1. 1000 permutations (RRPP).*

Table S2.12 – Pairwise comparisons among locomotor mode categories

All pairwise tests performed using the interaction model as full model and the additive model as null model, with 1000 RRPP permutations (95% confidence intervals). Dorsal view.

(a) Euclidean distances between group means in shape space (dist)

Comparison	d	UCL (95%)	Z	p
Hopper – Jumper	0.05911	0.11346	−0.7875	0.787
Hopper – Swimmer	0.05364	0.14315	−1.1334	0.858
Hopper – Walker	0.08194	0.18661	−0.9815	0.836
Jumper – Swimmer	0.04355	0.12713	−2.0290	0.977
Jumper – Walker	0.06916	0.16236	−0.8196	0.782
Swimmer – Walker	0.07679	0.19825	−1.4650	0.930

*Significance codes: 0 '***' 0.001 '**' 0.01 '*' 0.05 '.' 0.1 ' ' 1. 1000 permutations (RRPP).*

(b) Correlations between allometric vectors (VC)

Comparison	r	Angle	UCL (95%)	Z	p
Hopper – Jumper	0.9983	0.05898	0.10697	−0.5578	0.720
Hopper – Swimmer	0.9986	0.05351	0.13364	−0.9589	0.826
Hopper – Walker	0.9967	0.08176	0.17395	−0.7757	0.781
Jumper – Swimmer	0.9991	0.04353	0.11735	−1.7372	0.961
Jumper – Walker	0.9976	0.06905	0.14985	−0.6137	0.720
Swimmer – Walker	0.9971	0.07668	0.18486	−1.2324	0.892

*Significance codes: 0 '***' 0.001 '**' 0.01 '*' 0.05 '.' 0.1 ' ' 1. 1000 permutations (RRPP).*

(c) Differences in allometric vector lengths (DL)

Comparison	d	UCL (95%)	Z	p
Hopper – Jumper	0.002114	0.024378	−0.8731	0.798
Hopper – Swimmer	0.002193	0.035791	−1.0686	0.856
Hopper – Walker	0.000435	0.037328	−1.8543	0.961
Jumper – Swimmer	0.000079	0.026543	−2.5395	0.997
Jumper – Walker	0.001679	0.031471	−1.2259	0.889
Swimmer – Walker	0.001758	0.041502	−1.3399	0.905

*Significance codes: 0 '***' 0.001 '**' 0.01 '*' 0.05 '.' 0.1 ' ' 1. 1000 permutations (RRPP).*

(d) Within-group shape variances (var)

Observed variances by group — Hopper: 0.03578; Jumper: 0.03609; Swimmer: 0.03472; Walker: 0.03518.

Comparison	d	UCL (95%)	Z	p
Hopper – Jumper	0.000317	0.002617	−0.8905	0.815
Hopper – Swimmer	0.001057	0.003033	−0.0263	0.525
Hopper – Walker	0.000599	0.005624	−1.4003	0.910
Jumper – Swimmer	0.001374	0.003844	−0.0612	0.541
Jumper – Walker	0.000916	0.004503	−0.6800	0.744
Swimmer – Walker	0.000458	0.006541	−1.8626	0.959

*Significance codes: 0 '***' 0.001 '**' 0.01 '*' 0.05 '.' 0.1 ' ' 1. 1000 permutations (RRPP).*

Ventral view

Table S2.13 – Bilateral symmetry

Results of bilateral symmetry analysis for atlas shape in ventral view. n = 118 species.

Source	Df	SS	MS	R^2	F	Z	p
Individuals	388	14.5163	0.037413	0.93590	15.742	26.3826	0.001 ***
Side	1	0.0720	0.072032	0.00464	30.308	5.6088	0.001 ***
Individuals × Side	388	0.9221	0.002377	0.05945	—	—	—
Total	777	15.5105	—	—	—	—	—

*Significance codes: 0 '***' 0.001 '**' 0.01 '*' 0.05 '.' 0.1 ' ' 1. 1000 permutations (RRPP).*

Table S2.14 – Static allometry (OLS)

Multivariate regression of atlas shape on log CS estimated by OLS, without phylogenetic correction. Ventral view; n = 118 species.

Source	Df	SS	MS	R^2	F	Z	p
log(CS)	1	0.52408	0.52408	0.26984	42.869	6.2214	0.001 ***
Residuals	116	1.41814	0.01223	0.73016	—	—	—
Total	117	1.94222	—	—	—	—	—

*Significance codes: 0 '***' 0.001 '**' 0.01 '*' 0.05 '.' 0.1 ' ' 1. 1000 permutations (RRPP).*

Table S2.15 – Evolutionary allometry (PGLS)

Multivariate regression of atlas shape on log CS estimated by PGLS. Ventral view; n = 118 species.

Source	Df	SS	MS	R^2	F	Z	p
log(CS)	1	10.610	10.6104	0.18889	27.014	5.7560	0.001 ***
Residuals	116	45.562	0.3928	0.81111	—	—	—

Source	Df	SS	MS	R^2	F	Z	p
Total	117	56.172	—	—	—	—	—

*Significance codes: 0 '***' 0.001 '**' 0.01 '*' 0.05 '.' 0.1 ' ' 1. 1000 permutations (RRPP).*

Table S2.16 – Phylogenetic signal

Phylogenetic signal estimated for atlas shape and centroid size in ventral view. Results based on 1000 permutations.

Variable	Statistic	Value	p
Shape (Procrustes coordinates)	Z (effect size)	7.7405	0.001
Shape (Procrustes coordinates)	λ (Pagel's lambda)	1	—
Shape (Procrustes coordinates)	K (Brownian Motion ratio)	0.0469	—
Centroid size	K (Blomberg's K)	0.0641	0.001

*Significance codes: 0 '***' 0.001 '**' 0.01 '*' 0.05 '.' 0.1 ' ' 1. 1000 permutations (RRPP).*

Table S2.17 – Effect of microhabitat on atlas shape (additive PGLS model)

PGLS additive model testing the effects of log CS and microhabitat on atlas shape. Ventral view; n = 118 species.

Source	Df	SS	MS	R^2	F	Z	p
log(CS)	1	10.610	10.6104	0.18889	27.6111	5.8088	0.001 ***
Microhabitat	3	2.138	0.7126	0.03806	1.8543	1.5230	0.065 .
Residuals	113	43.424	0.3843	0.77305	—	—	—
Total	117	56.172	—	—	—	—	—

*Significance codes: 0 '***' 0.001 '**' 0.01 '*' 0.05 '.' 0.1 ' ' 1. 1000 permutations (RRPP).*

Table S2.18 – Heterogeneous allometry by microhabitat (interaction PGLS model)

PGLS interaction model testing whether allometric trajectories differ among microhabitat categories. Ventral view; n = 118 species.

Source	Df	SS	MS	R^2	F	Z	p
log(CS)	1	10.610	10.6104	0.18889	28.4490	5.8295	0.001 ***
Microhabitat	3	2.138	0.7126	0.03806	1.9106	1.5854	0.053 .
log(CS) × Microhabitat	3	2.398	0.7993	0.04269	2.1431	1.8261	0.034 *
Residuals	110	41.026	0.3730	0.73036	—	—	—
Total	117	56.172	—	—	—	—	—

*Significance codes: 0 '***' 0.001 '**' 0.01 '*' 0.05 '.' 0.1 ' ' 1. 1000 permutations (RRPP).*

Table S2.19 – Nested model comparison: microhabitat

Nested ANOVA comparing the additive and interaction models for microhabitat. Ventral view.

Model	Res. Df	Df	RSS	SS	MS	R^2	F	Z	p
shape ~ log(CS) + Microhabitat	113	1	43.424	—	—	0.000000	—	—	—
shape ~ log(CS) × Microhabitat	110	3	41.026	2.3979	0.79931	0.042689	2.1431	1.8261	0.034

*Significance codes: 0 '***' 0.001 '**' 0.01 '*' 0.05 '.' 0.1 ' ' 1. 1000 permutations (RRPP).*

Table S2.20 – Pairwise comparisons among microhabitat categories

All pairwise tests with 1000 RRPP permutations (95% confidence intervals). Ventral view.

(a) Euclidean distances between group means (dist)

Comparison	d	UCL (95%)	Z	p
Aquatic – Arboreal	0.05165	0.15141	−1.6006	0.950
Aquatic – Semi-aquatic	0.10429	0.18476	−0.9046	0.814
Aquatic – Terrestrial	0.05198	0.13429	−1.2150	0.895
Arboreal – Semi-aquatic	0.10419	0.17304	−0.2944	0.602
Arboreal – Terrestrial	0.06640	0.13046	−0.4069	0.663
Semi-aquatic – Terrestrial	0.05928	0.10323	−0.7094	0.762

*Significance codes: 0 '***' 0.001 '**' 0.01 '*' 0.05 '.' 0.1 ' ' 1. 1000 permutations (RRPP).*

(b) Correlations between allometric vectors (VC)

Comparison	r	Angle	UCL (95%)	Z	p
Aquatic – Arboreal	0.9987	0.05162	0.14508	−1.4730	0.935
Aquatic – Semi-aquatic	0.9946	0.10403	0.17573	−0.6772	0.747
Aquatic – Terrestrial	0.9987	0.05195	0.12825	−1.0779	0.864
Arboreal – Semi-aquatic	0.9946	0.10391	0.16352	−0.1047	0.527
Arboreal – Terrestrial	0.9978	0.06635	0.12183	−0.2585	0.600
Semi-aquatic – Terrestrial	0.9983	0.05904	0.09851	−0.4806	0.688

*Significance codes: 0 '***' 0.001 '**' 0.01 '*' 0.05 '.' 0.1 ' ' 1. 1000 permutations (RRPP).*

(c) Differences in allometric vector lengths (DL)

Comparison	d	UCL (95%)	Z	p
Aquatic – Arboreal	7.98×10^{-4}	0.029456	−1.6378	0.940
Aquatic – Semi-aquatic	4.07×10^{-3}	0.044628	−1.0297	0.824
Aquatic – Terrestrial	7.14×10^{-4}	0.025846	−1.5654	0.931
Arboreal – Semi-aquatic	3.27×10^{-3}	0.038085	−1.0575	0.845
Arboreal – Terrestrial	8.46×10^{-5}	0.025776	−2.3973	0.993
Semi-aquatic – Terrestrial	3.35×10^{-3}	0.025699	−0.8277	0.778

*Significance codes: 0 '***' 0.001 '**' 0.01 '*' 0.05 '.' 0.1 ' ' 1. 1000 permutations (RRPP).*

(d) Within-group shape variances (var)

Observed variances by group — Aquatic: 0.03026; Arboreal: 0.02950; Semi-aquatic: 0.02926; Terrestrial: 0.02828.

Comparison	d	UCL (95%)	Z	p
Aquatic – Arboreal	0.000761	0.002446	−0.0868	0.541
Aquatic – Semi-aquatic	0.001003	0.002268	0.3988	0.364
Aquatic – Terrestrial	0.001982	0.003086	0.6214	0.283
Arboreal – Semi-aquatic	0.000242	0.001948	−0.8997	0.798
Arboreal – Terrestrial	0.001221	0.002428	0.4221	0.362
Semi-aquatic – Terrestrial	0.000979	0.001892	0.2897	0.400

*Significance codes: 0 '***' 0.001 '**' 0.01 '*' 0.05 '.' 0.1 ' ' 1. 1000 permutations (RRPP).*

Table S2.21 – Effect of locomotor mode on atlas shape (additive PGLS model)

PGLS additive model testing the effects of log CS and locomotor mode on atlas shape. Ventral view; n = 118 species.

Source	Df	SS	MS	R^2	F	Z	p
log(CS)	1	10.610	10.6104	0.18889	26.7750	5.7551	0.001 ***
Locomotor mode	3	0.782	0.2606	0.01392	0.6575	−0.4751	0.680
Residuals	113	44.780	0.3963	0.79719	—	—	—
Total	117	56.172	—	—	—	—	—

*Significance codes: 0 '***' 0.001 '**' 0.01 '*' 0.05 '.' 0.1 ' ' 1. 1000 permutations (RRPP).*

Table S2.22 – Heterogeneous allometry by locomotor mode (interaction PGLS model)

PGLS interaction model testing whether allometric trajectories differ among locomotor categories. Ventral view; n = 118 species.

Source	Df	SS	MS	R^2	F	Z	p
log(CS)	1	10.610	10.6104	0.18889	27.5654	5.7781	0.001 ***
Locomotor mode	3	0.782	0.2606	0.01392	0.6769	−0.4257	0.666
log(CS) × Locomotor mode	3	2.439	0.8129	0.04342	2.1120	1.5631	0.060 .
Residuals	110	42.341	0.3849	0.75377	—	—	—
Total	117	56.172	—	—	—	—	—

*Significance codes: 0 '***' 0.001 '**' 0.01 '*' 0.05 '.' 0.1 ' ' 1. 1000 permutations (RRPP).*

Table S2.23 – Nested model comparison: locomotor mode

Nested ANOVA comparing the additive and interaction models for locomotor mode. Ventral view.

Model	Res. Df	Df	RSS	SS	MS	R^2	F	Z	p
shape ~ log(CS) + Locomotor mode	113	1	44.780	—	—	0.000000	—	—	—
shape ~ log(CS) × Locomotor mode	110	3	42.341	2.4388	0.81294	0.043417	2.1120	1.5631	0.060

*Significance codes: 0 '***' 0.001 '**' 0.01 '*' 0.05 '.' 0.1 ' ' 1. 1000 permutations (RRPP).*

Table S2.24 – Pairwise comparisons among locomotor mode categories

All pairwise tests with 1000 RRPP permutations (95% confidence intervals). Ventral view.

(a) Euclidean distances between group means (dist)

Comparison	d	UCL (95%)	Z	p
Hopper – Jumper	0.07552	0.14697	−0.6609	0.740
Hopper – Swimmer	0.06072	0.16665	−1.1845	0.883
Hopper – Walker	0.06582	0.18285	−1.2738	0.894
Jumper – Swimmer	0.04987	0.15229	−1.1314	0.870
Jumper – Walker	0.08221	0.14892	−0.1376	0.553
Swimmer – Walker	0.05572	0.20658	−1.9499	0.976

*Significance codes: 0 '***' 0.001 '**' 0.01 '*' 0.05 '.' 0.1 ' ' 1. 1000 permutations (RRPP).*

(b) Correlations between allometric vectors (VC)

Comparison	r	Angle	UCL (95%)	Z	p
Hopper – Jumper	0.9972	0.07537	0.13964	−0.4873	0.675
Hopper – Swimmer	0.9982	0.06057	0.15747	−1.0454	0.855
Hopper – Walker	0.9978	0.06565	0.16932	−1.1404	0.869
Jumper – Swimmer	0.9988	0.04985	0.14396	−1.0120	0.840
Jumper – Walker	0.9966	0.08210	0.14257	0.0114	0.491
Swimmer – Walker	0.9985	0.05562	0.19417	−1.8302	0.966

*Significance codes: 0 '***' 0.001 '**' 0.01 '*' 0.05 '.' 0.1 ' ' 1. 1000 permutations (RRPP).*

(c) Differences in allometric vector lengths (DL)

Comparison	d	UCL (95%)	Z	p
Hopper – Jumper	0.002421	0.034080	−1.1384	0.864
Hopper – Swimmer	0.002579	0.033868	−1.1092	0.871
Hopper – Walker	0.000662	0.037640	−1.8231	0.965
Jumper – Swimmer	0.000158	0.027278	−2.3921	0.990
Jumper – Walker	0.001760	0.028239	−1.0780	0.847

Comparison	d	UCL (95%)	Z	p
Swimmer – Walker	0.001917	0.039128	−1.3099	0.908

*Significance codes: 0 '***' 0.001 '**' 0.01 '*' 0.05 '.' 0.1 ' ' 1. 1000 permutations (RRPP).*

(d) Within-group shape variances (var)

Observed variances by group — Hopper: 0.02928; Jumper: 0.02618; Swimmer: 0.02747; Walker: 0.02568.

Comparison	d	UCL (95%)	Z	p
Hopper – Jumper	0.003100	0.003665	1.2204	0.104
Hopper – Swimmer	0.001805	0.002761	0.9732	0.164
Hopper – Walker	0.003598	0.003292	1.7191	0.036 *
Jumper – Swimmer	0.001295	0.004072	−0.3742	0.662
Jumper – Walker	0.000498	0.002951	−0.3840	0.651
Swimmer – Walker	0.001792	0.003798	0.4057	0.361

*Significance codes: 0 '***' 0.001 '**' 0.01 '*' 0.05 '.' 0.1 ' ' 1. 1000 permutations (RRPP).*
